# Genome-wide prediction of cis-regulatory regions using supervised deep learning methods

**DOI:** 10.1186/s12859-018-2187-1

**Published:** 2018-05-31

**Authors:** Yifeng Li, Wenqiang Shi, Wyeth W. Wasserman

**Affiliations:** 1 Centre for Molecular Medicine and Therapeutics, BC Children’s Hospital Research Institute, Department of Medical Genetics, University of British Columbia, Rm 3109, 950 West 28th Avenue, Vancouver, V5Z 4H4 Canada; 2 Digital Technologies Research Centre, National Research Council Canada, Building M-50, 1200 Montreal Road, Ottawa, K1A 0R6 Canada

**Keywords:** *cis*-regulatory region, Enhancer, Promoter, Deep learning

## Abstract

**Background:**

In the human genome, 98% of DNA sequences are non-protein-coding regions that were previously disregarded as junk DNA. In fact, non-coding regions host a variety of *cis*-regulatory regions which precisely control the expression of genes. Thus, Identifying active *cis*-regulatory regions in the human genome is critical for understanding gene regulation and assessing the impact of genetic variation on phenotype. The developments of high-throughput sequencing and machine learning technologies make it possible to predict *cis*-regulatory regions genome wide.

**Results:**

Based on rich data resources such as the Encyclopedia of DNA Elements (ENCODE) and the Functional Annotation of the Mammalian Genome (FANTOM) projects, we introduce DECRES based on supervised deep learning approaches for the identification of enhancer and promoter regions in the human genome. Due to their ability to discover patterns in large and complex data, the introduction of deep learning methods enables a significant advance in our knowledge of the genomic locations of *cis*-regulatory regions. Using models for well-characterized cell lines, we identify key experimental features that contribute to the predictive performance. Applying DECRES, we delineate locations of 300,000 candidate enhancers genome wide (6.8% of the genome, of which 40,000 are supported by bidirectional transcription data), and 26,000 candidate promoters (0.6% of the genome).

**Conclusion:**

The predicted annotations of *cis*-regulatory regions will provide broad utility for genome interpretation from functional genomics to clinical applications. The DECRES model demonstrates potentials of deep learning technologies when combined with high-throughput sequencing data, and inspires the development of other advanced neural network models for further improvement of genome annotations.

**Electronic supplementary material:**

The online version of this article (10.1186/s12859-018-2187-1) contains supplementary material, which is available to authorized users.

## Background

In this article, we apply deep supervised analysis methods to identify the positions of active *cis*-regulatory regions (CRRs), including both enhancers and promoters, across the human genome. CRRs play a crucial role in precise control of gene expression. Promoters and enhancers act via complex interactions across time and space in the nucleus to control when, where and at what magnitude genes are active. CRRs, through interactions with proteins such as histones and sequence-specific DNA-binding transcription factors (TFs), help specify the formation of diverse cell types and respond to changing physiological conditions. While gene expression is ultimately a reflection of regulation across multiple processes, the key role of promoters and enhancers has been a central focus of genome annotation for the past decade. The investment in generating informative data for the detection of these regions has been immense, in part motivated by the anticipation that advanced computational approaches would be able to transform the data into a reliable annotation of the genome.

Promoters and enhancers were early discoveries during the molecular characterization of genes. While promoters specify and enable the positioning of RNA polymerase machinery at transcription initiation sites, enhancers modulate the activity of promoters from linearly distal locations away from transcript initiation sites [1, 2]. The delineation between the classes has become increasingly challenging, with some literature suggesting the two categories are the edges of a continuous spectrum of CRRs [3]. Indeed, it has long been observed that sequences flanking transcription initiation regions can function as enhancers (promoter-proximal regions), and in recent years, it has been observed that there are transcripts initiated at the edges of active enhancers [4, 5]. For the purpose of this report, we address the two as distinct classes, but discuss the relationship between our findings and the continuous class model.

The use of computational methods to detect the locations of promoters and enhancers has been a key focus of bioinformatics for twenty years (see reviews [6, 7]). With the advances of experimental procedures for profiling the properties of chromatin and RNA transcripts, a new wave of methods has arrived. Given the small set of reliable enhancer annotations, it was appropriate that the first among these methods used unsupervised learning. For instance, both ChromHMM [8] and Segway [9] segment the genome into sequence classes based on ENCODE project data [10], such as histone modification ChIP-seq (chromatin immunoprecipitation followed by sequencing [11]) signals. Such unsupervised methods infer hidden states based on observed signals, and then associate an element to each hidden state. The states are subsequently labelled with biological functions based on enrichment for known examples. A test of predicted Enhancers for the K562 leukemia cell line by the Combined method (unifying ChromHMM and Segway annotations) [12] using a high-throughput reporter gene assay [13] revealed that only 26% of predicted enhancers have regulatory activity [14]. The assessment showed that the predicted Weak Enhancers, a class associated with lower H3K27ac and H3K36me3 signals, unexpectedly drove higher gene expression than the predicted Enhancers. It is evident that improvements are needed, potentially involving the use of additional experimental features and alternative machine learning approaches.

Despite the limited set of precisely annotated active enhancers, supervised machine learning models have been attempted to predict enhancer regions. In each case, a distinct definition of a suitable positive training set of enhancers was taken. A random-forest method was used in [15] to classify TF bound regions with a focus on observed binding patterns, generating sets of two-class classifiers to distinguish regions based on binding activity and position relative to promoter regions. A random-forest based enhancer classification method was devised in [16] with histone modification ChIP-seq data as features, using p300 bound regions as the basis for training. An AdaBoost-based model was proposed in [17] for the prediction of enhancers that are defined by p300 binding sites overlapping with DNase-I hypersensitive sites and distal to annotated TSS. Chen et al. applied multinomial logistic regression with LASSO regularization to find key features for the classification of stem cell-specific functional enhancer regions [18]. Using STARR-seq data, a new experimental approach for screening candidate enhancer sequences [19], dinucleotide repeat motifs (DRMs) were found to be enriched in broadly active enhancers, leading to a proposition that a small set of TF binding site motifs and DRMs might be sufficient for enhancer prediction [20].

New laboratory methods are emerging, providing a refined resolution of CRR locations. The majority of human DNA is transcribed, producing diverse types of RNA. In particular, transcripts generated at the edges of enhancers, enhancer RNAs (eRNAs), allow for the experimental readout of active regulatory regions. Global run-on and sequencing (GRO-seq) protocols [21] measure the 5’-end of nascent RNAs revealing the divergent transcriptional signature of both transcriptionally active promoters and enhancers [5]. Using GRO-seq signals, a support vector regression model (dReg) was developed to predict active transcriptional regulatory elements [22]. The cap analysis of gene expression (CAGE) technique [23] captures the 5’-end of RNA transcripts, enabling a precise determination of transcript initiation sites. Using CAGE, the FANTOM5 Consortium has identified an atlas of transcriptionally active promoters [24] and a permissive set of 43,011 transcriptionally active enhancers characterized by bidirectional eRNAs [4] across hundreds of human cell types and tissues. These enhancers were validated with high success rates ranging from 67.4 to 73.9% [4]. Compared to protein-coding RNAs, eRNAs are believed to degenerate quickly, and only a small number of tissues have been explored with sufficient depth to reveal eRNAs. While the FANTOM enhancer set is therefore incomplete, it provides a uniquely large inventory of high-quality enhancers to use for the training of machine learning approaches. An ensemble support vector machine method suggested the potential to distinguish enhancers based on such data [25].

We have previously proposed and herein present the use of a deep feature selection (DFS) model for the supervised prediction of CRRs [26]. Deep learning is a dramatic advance in the frontier of artificial intelligence [27–29]. Unlike widely used linear models, deep learning approaches model complex systems and capture high-level knowledge from data. Driven by big and rich data, deep learning has been successfully applied in various areas such as automatic image annotation and speech language processing [30]. Bioinformaticians have started using this powerful tool for next-generation sequencing data mining, such as predicting the impact of variations on exon splicing [31] and the effects of noncoding variants on chromatin [32], detecting TF binding patterns [33], and predicting protein secondary structures [34].

Our study stands on three important legs. First, the precisely annotated FANTOM promoters and enhancers, which provide the largest experimentally defined collection of CRRs. Second, the ENCODE project genome-wide feature data, such as histone modifications, TF binding, RNA transcripts, chromatin accessibility, and chromatin interactions. Third, deep learning methods to distinguish CRRs based on the available data. We unite the three components to create the DECRES (DEep learning for identifying Cis-Regulatory ElementS and other applications) model, with which we identify the most comprehensive collection of CRRs across the human genome yet compiled.

## Results

### Deep learning accurately distinguishes active enhancers and promoters from background

**Fig. 1:**
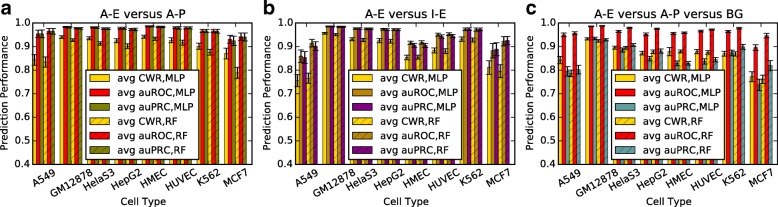
Mean performance and standard deviation of 100 runs using the MLP model on our respectively sampled train-test partitions of eight cell types. **a** Classification performances of A-E versus A-P. **b** Classification performances of A-E versus I-E. **c** Classification performances of A-E versus A-P versus BG. MLP: Multilayer Perception, RF: Random Forest, A-E: Active Enhancer, A-P: Active Promoter, A-X: Active Exon, I-E: Inactive Enhancer, I-P: Inactive Promoter, I-X: Inactive Exon, UK: Unknown or Uncharacterized, BG: I-E+I-P+A-X+I-X+UK

We investigated the capacity of deep learning models to separate enhancers and promoters, and to distinguish them from other regions and between activity states. We trained a deep feedforward neural network over our balanced labelled training sets to predict our (unbalanced) test sets from each well-characterized cell type, repeating the procedure 100 times. The deep model takes experimentally derived features over genomic regions as inputs and outputs class labels of these regions with probabilities (see Additional file 1: Table S1 for the total number of samples of each class and Additional file 1: Table S2 for the number of available features; see Methods). For narrative convenience, hereafter we refer to active enhancer, active promoter, active exon, inactive enhancer, inactive promoter, inactive exon, and unknown (or uncharacterized) region as A-E, A-P, A-X, I-E, I-P, I-X, and UK, respectively. Under the assumption that active CRRs are undergoing transcription, active applies to regions in which CAGE transcript initiation events are observed in the tissue of focus, while inactive refers to regions detected in other tissues, but not in the focus tissue. We recorded the mean class-wise rate (i.e. averaged sensitivities of all classes), area under the receiver operating characteristic curve (auROC), and the area under the precision-recall curve (auPRC) in Fig. 1 and Additional file 1: Figure S1.

There are four aspects of the results that we highlight, which affirm the capacity of our supervised deep learning approach to distinguish between classes of CRRs and background. First, we are able to distinguish between active enhancers and promoters (A-E versus A-P) (Fig. 1a). We used A-E and A-P as positive and negative training classes, respectively. Overall, we found that A-E and A-P are highly separable. Second, we can distinguish active and inactive CRRs (either enhancers or promoters). From Fig. 1b and Additional file 1: Figure S1A, it can be observed that mean auPRCs on GM12878, HelaS3, HepG2, and K562, which have the largest training sets, are above 0.95 with small variances for both enhancers and promoters. In the rest of this paper, we exclude A549 and MCF7 cell lines in most analyses due to limited data availability. Third, not unexpectedly, it is difficult to distinguish between inactive enhancers and promoters (Additional file 1: Figure S1B). Seven of the mean class-wise rates for the eight cell types were lower than 0.80. While there are some indications that a portion of inactive promoters have some machinery present, it was our expectation that such regions will largely not exhibit strong transcription factor binding or appropriate epigenetic signatures to inform a model. Fourth, we tested the applicability of predicting A-E and A-P from the super background (BG) class merging I-E, I-P, A-X, I-X, and UK (Fig. 1c). The results on six cell types were promising, all exceeded 0.80 auPRC. If A-E and A-P are merged further to form a super class (A-E+A-P), higher performance is achieved (Additional file 1: Figure S1C). All auPRCs on these six cell types went beyond 0.89 auPRC. Furthermore, we also tested a random forest method, another state-of-the-art classifier, on our labelled data. Similar performance was obtained on all six experimental settings. The random forest method exhibited slightly better performance for A549 and MCF7 datasets, which both have low numbers of enhancers. In expectation that more annotated enhancers are becoming available, we will continue using MLP and exploring other deep learning approaches such as convolutional neural networks and recurrent neural networks.

### DECRES gives higher sensitivity and precision on FANTOM annotated regions

**Fig. 2:**
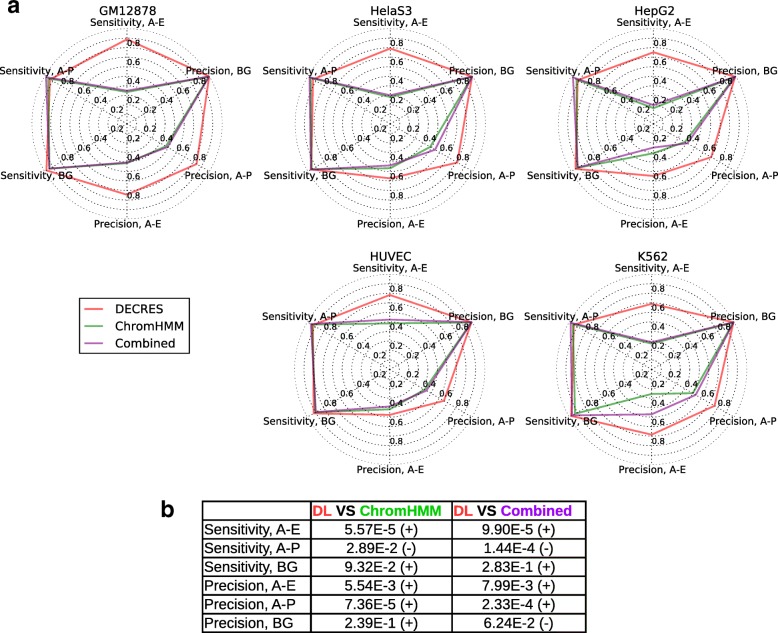
Comparison of the supervised method (DECRES) and unsupervised methods (ChromHMM and Combined) on five FANTOM annotated test sets in radar charts (**a**) and significance tests (**b**). The ENCODE segmentations were downloaded from [66]. We relabelled the annotations of ChromHMM and Combined. For ChromHMM segmentations, the Tss, TssF, and PromF classes were merged to A-P; the Enh, EnhF, EnhW, EnhWF classes were merged to A-E; and the rest were denoted by BG. When processing the Combined annotations, TSS and PF were relabelled to A-P; E and WE were relabelled to A-E; and the rest to BG. The *p*-values in (**b**) were obtained from two-tailed Student’s t-test on all cell types. The signs of statistic values are indicated in brackets

To assess the relative utility of our supervised deep method for CRR prediction, we compared it with the unsupervised ChromHMM and ChromHMM-Segway Combined methods [8, 12] using FANTOM annotations on five available cell types as reference. They were compared on unbalanced sets reflecting the true genomic background. The results are compared in Fig. 2a which displays radar charts where the larger and more convex the area is, the better the performance. It is intuitive that supervised approaches are preferred when labelled training data is sufficient. Furthermore, both unsupervised methods were developed prior to public release of the FANTOM5 data and are therefore at a disadvantage. However, these annotations are widely used by the community and hence the relative performance of DECRES to the standard is of interest. Overall, we observe that DECRES outperforms ChromHMM and Combined methods which in turn deliver similar performance. These unsupervised methods consistently have lower sensitivities for active enhancer detection (*p* = 5.57E-5 and 9.90E-5 for DECRES versus ChromHMM and Combined respectively, two tailed Student’s t-test; see Fig. 2b) and lower precision for active promoter detection (*p*=7.36E-5 and 2.33E-4 for DECRES versus ChromHMM and Combined respectively, two tailed Student’s t-test; see Fig. 2b). Using ChromHMM, the active enhancer sensitivity ranges from 16.5% to 48.4% (numbers are consistent with the test on ENCODE predicted enhancers reported in [14]), while our deep model ranges from 69% (K562) to 88.8% (GM12878). Moreover, ChromHMM achieves a maximum precision of 49.8% for active promoter prediction, while the maximum for DECRES is of 84.3%.

### Evaluation of DECRES performance with independent experimental data

As the initial evaluation focused on FANTOM eRNA-based annotation of CRRs, the type of data used to train our supervised model, we sought to assess performance on data generated by alternative methods. We identified two independent collections of laboratory validated enhancers to further assess the performance of DECRES: a CRE-seq collection of regions tested in K562 cells [14] and MPRA (massively parallel reporter assay) collections tested in K562 and HepG2 cells [35]. In both instances, the set of regions that fail to direct expression may be falsely predicted by the assessed methods, but may also reflect the facts that the experimental procedures only include a small segment of regulatory DNA and that plasmid-based assays do not recapitulate chromatin properties. Given the nature of the data, we anticipate a portion of the experimental negatives to be *bona fide* regulatory regions.

In the first independent set, subsets of predicted K562 enhancers and negative regions (as predicted by the Combined ChromHMM and Segway method) were assessed in the laboratory using CRE-seq [14]. In that study, only 33% of the “Combined” predicted regulatory regions were found to be positive in the experiment, compared to 7% for the negative set. Using DECRES trained on all available active regulatory regions of K562 cells, we therefore validated our method on 386 regions showing active enhancer activity in K562 as validated by CRE-seq compared to the 298 control regions (Additional file 1: Table S3). Highly consistent with the results above, a sensitivity of 65.5% (254/386) for the experimentally validated regions were successfully predicted as A-E; the remaining 132 regions were predicted as background (none were classified as promoters). For the 812 tested predictions that were inactive in the CRE-seq experiment, DECRES classified 53.3% (433/812) as positive. For the 298 negative control regions, DECRES predicted all to be negative (including the 16 that were active in the CRE-seq experiment). Importantly, as the DECRES scores rise, the quality of the predictions increase. We drew the histogram of DECRES membership scores of 254 and 433 experimentally positive and negative Combined enhancers that were predicted as A-Es by DECRES (Additional file 1: Figure S2). The distributions are significantly different (*p* = 0.014, two-sided Mann-Whitney rank test).

The second independent collection, in which K562 and HepG2-specific “strong enhancer” (as predicted by ChromHMM) containing predicted TF binding sites for cell-selective TFs were tested using a massively parallel reporter assay (MPRA) [35]. Only 41% of the enhancers were detected to be significantly expressed (*p* = 0.05, two-sided Mann-Whitney rank test). We used DECRES to predict the classes of the MPRA positive and MPRA negative enhancers. Our result in Additional file 1: Table S3 shows that 98.4% (120/122) and 97.8% (182/186) of the MPRA positive enhancers were respectively predicted to be A-Es by DECRES for K562 and HepG2 cells, while 92.3% (179/194) and 81.3% (217/267) of the MPRA negative enhancers were still predicted as A-Es for K562 and HepG2, respectively, but with different distributions of DECRES scores (*p*= 4.8E-6 and *p*= 2.3E-6 for K562 and HepG2 respectively, two-sided Mann-Whitney rank test) (Additional file 1: Figure S2). Consistent with the other independent data, the higher the DECRES scores the more likely they are to be positive.

### Assessing the utility of DNA sequence properties on the performance of DECRES

**Fig. 3:**
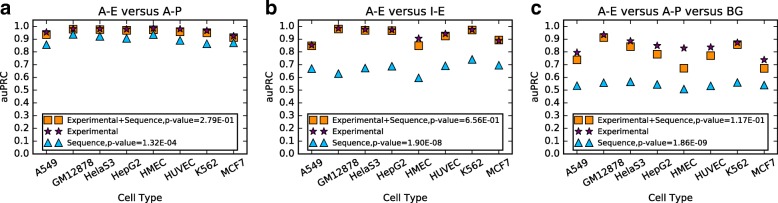
Comparing the mean auPRCs over 100 resampling and retraining on our labelled regions using different feature sets. “Experimental” means our experimentally derived next generation sequencing feature set. “Sequence” means the set of 351 sequence properties used in [25]. “Experimental+Sequence” means the combination of these two sets. **a**. Comparison of the three feature sets in A-E versus A-P. **b**. Comparison of the three feature sets in A-E versus I-E. **c**. Comparison of the three feature sets in A-E versus A-P versus BG. The *p*-values in each legend were obtained using two-tailed Student’s t-test to compare “Experimental”-based results with “Experimental+Sequence”-based and “Sequence”-based results, respectively

Recent studies confirmed that DNA sequence properties can be useful for the recognition of promoters and enhancers [3, 5, 25], and the discrimination between active and inactive regulatory sequences [36, 37] using string sequence kernels. This builds on the long-recognized capacity for the inclusion of CpG islands as features to improve promoter prediction [38]. We sought to determine if DNA sequence features can be informative to distinguish between promoters and enhancers, and between active and inactive classes. We trained the model with 351 sequence features (originally used in [25]) in multiple scenarios. Results are displayed in Fig. 3 and Additional file 1: Figure S3. First, a deep method restricted to sequence features for discriminating A-E and A-P (Fig. 3a) delivered auPRCs from 0.8567 to 0.9370, confirming that sequence attributes are indeed informative. Second, sequence features have a limited utility for distinguishing between active and inactive states of enhancers and promoters, which is logical; while the experimentally derived features could highly separate them (*p*=1.90E-08 and 5.06E-08 for enhancers and promoters respectively, two-tailed Student’s t-test; see Fig. 3b and Additional file 1: Figure S3A). Using sequence features in the absence of experimental features has a lower performance in classifying A-E, A-P and BG across all eight cell types (*p*=1.86E-09, two-tailed Student’s t-test; see Fig. 3c). Finally, better results were not achieved by combining experimental and sequence features (*p*=2.79E-01, 6.56E-01 and 1.17E-01 in Fig. 3, two-tailed Student’s t-test).

### Key features for DECRES performance

As experimental data can be time consuming and expensive to produce, we sought to determine the minimal set of features most informative for CRR prediction from a computational perspective. We used randomized deep feature selection (randomized DFS or RDFS) and random forest (RF) models (see Methods) for two-class [A-E+A-P (or CRR) versus BG] and three-class (A-E versus A-P versus BG) classifications on four cell types (GM12878, HelaS3, HepG2, and K562) which have 72-135 features available.

**Fig. 4:**
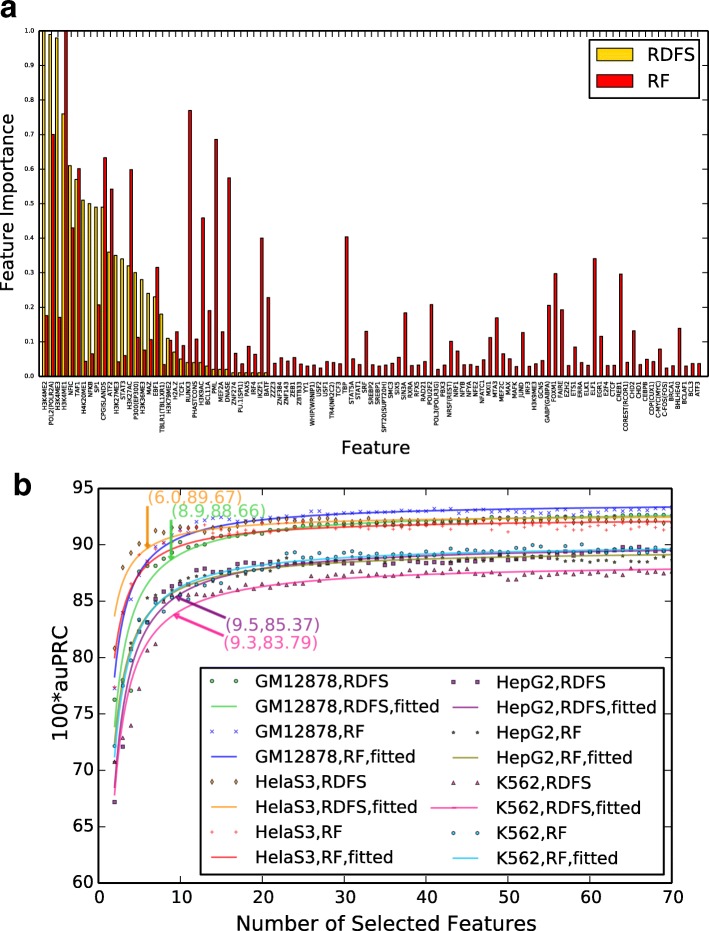
Feature importance and classification performance in the 3-class (A-E versus A-P versus BG) scenario. **a** Feature importance discovered by randomized DFS (RDFS) and random forest (RF) on GM12878. The random forest’s feature importance scores were normalized to [0,1] for better comparison with randomized DFS. **b** auPRC versus the number of features incorporated into the RDFS and RF. The annotated points indicate where a line with slope 0.5 intersects a fitted curve

Figure 4a and Additional file 1: Figure S4A display the feature importance scores discovered by randomized DFS and random forest for the three-class classification. The feature importance scores produced by these methods should be interpreted differently. Similar to a forward selection, the feature importance scores from randomized DFS reflect which features are preferred in the early stage of the sparse model, while the importance score of a feature by random forest indicates the role of this feature in the context of its use with all other features. Thus, using both methods in this study enables us to gain different insights into the data. In our experiments, both methods can capture the most important features as indicated by importance scores across all four cell lines. For example, both methods agree that Pol2, H3K4me1, Taf1, and H3K27ac are useful for distinguishing active enhancers and promoters from the background in GM12878 cell line. In some cases, the different measures complement each other. For instance, H3K4me2 and H4K20me1 are marked as key features by the randomized DFS, which is convincing as indicated by the box plots in Additional file 1: Figure S4B and Figure S6-S13, but are overlooked by random forest. Tbp was highlighted by random forest in GM12878 and HelaS3 cells, but was not picked up by randomized DFS. Examining the box plots of this feature in Additional file 1: Figures S6 and S7 reveals that this feature is discriminative to distinguish active enhancers and promoters from background, but there is not a dramatic difference between active enhancers and promoters. Important features incorporated into a random forest model may not be incorporated until a latter stage of the DFS process. For instance, in K562 cell line, C-Myc was emphasized by random forest, which is indeed reasonable as shown in Additional file 1: Figure S12 and was not selected as an initial feature in the DFS process.

For the development of machine learning methods in genome annotation, minimizing the number of features required decreases cost and increases the capacity for biological interpretation. Figure 4b and Additional file 1: Figure S5B show the changes of test auPRCs as the numbers of selected features increase for the three-class and two-class classifications, respectively. In both cases, test auPRCs increase dramatically for the initial features, then performance plateaus. Comparing the randomized DFS curves with the random forest curves, we can see that there is no single optimal curve. A few key features are sufficient for a good prediction performance. To define an optimal number of features needed, we fit the curves in Fig. 4b and Additional file 1: Figure S5B and selected the intersection point for a line with slope of 0.5 on the randomized DFS curves (see Methods). Fewer features are needed for two-class CRR prediction (6 features) compared to three-class models intended to distinguish between A-E, A-P and background (10 features).

The distributions of the top ten features for three-class predictions (A-E, A-P, and BG) are given in Additional file 1: Figure S4B. Using the top ten features for each cell, auPRCs of 0.9022, 0.9156, 0.8651, and 0.8565 were achieved on GM12878, HelaS3, HepG2, and K562, respectively. Half of these top features are histone modifications, of which H3K4me1, H3K4me2, H3K4me3, and H3K27me3 were commonly selected features for the three-class models, in agreement with existing knowledge [2, 3, 39, 40]. Among transcription factors (including co-factors), Taf1 and p300, as well as RNA polymerase II (Pol2), are frequently selected, which is also consistent with existing knowledge [41, 42].

Additional file 1: Figure S5C shows box plots of the top six selected features by randomized DFS for two-class predictions. Using these features, auPRCs of 0.9561, 0.9627, 0.926, and 0.9555 were obtained on the four cell types, respectively. For most features, the ranges of values are elevated in A-E and A-P relative to the background categories. Half of the selected features are DNase-seq and histone modification ChIP-seq data including H3K4me2, H3K27ac, and H3K27me3. The box plots of these features indicate that they distinguish A-E and A-P from background [2, 39, 40].

### The majority of DECRES’s genome-wide predictions are supported by other methods

We trained 2- and 3-class multilayer perceptron (MLP) models (see Methods) using all reference (labelled) data for training, in order to predict CRRs across the entire genome for six cell types (A549 and MCF7 were excluded). The 2-class model identified 227,332 CRRs (adjacent regions were merged), which occupy 4.8% of the genome (Additional file 1: Table S4). A total of 9153 CRRs were ubiquitously predicted across all six cell types. For the 3-class prediction, we obtained 301,650 A-E regions (6.8% of the genome) and 26,555 A-P regions (0.6% of the genome) together with 11,886 ubiquitous A-Es and 3678 ubiquitous A-Ps. The genome-wide predictions for all six cell types are available in Additional file 2.

**Fig. 5:**
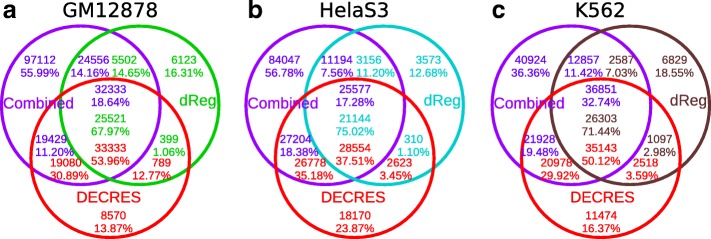
Agreements of the DECRES CRRs with the Combined and dReg CRRs on three cell types (**a**: GM12878, **b**: HelaS3, **c**: K562), respectively. The TSS, PF, E, and WE segmentations from Combined were relabelled to CRRs. The active transcriptional regulatory elements (TREs) predicted by dReg were renamed to CRRs

Next, we examined the overlap of our predicted CRRs with the Combined [12] and dReg [22] predictions on GM12878, HelaS3, and K562. The majority of CRRs predicted by DECRES overlap with the results from either Combined or dReg, specifically 86.13%, 76.13%, and 83.63% for GM12878, HelaS3, and K562, respectively (Fig. 5). A subset (13.87% on GM12878, 23.87% on HelaS3, and 16.37% on K562) of DECRES predictions do not overlap with predictions from the other two tools. Notably, a large portion of the Combined predictions (56.78% on HelaS3, 55.99% on GM12878, and 36.36% on K562) do not overlap with those from the supervised methods, which is consistent with its low observed validation rate [14]. Furthermore, DECRES predictions tend to have a finer resolution for both A-P and A-E regions (see Additional file 1: Figure S14 for an example).

We investigated how many among our genome-wide predictions are supported by the VISTA enhancer set [43]. Despite the fact that the majority of the VISTA enhancers are extremely conserved across development, we still find that 37.1% (850/2,293) of experimentally confirmed and unconfirmed VISTA enhancers overlap with the predicted A-Es, while merely 4.8% (110/2,293) of these VISTA enhancers overlap with the predicted A-Ps. Results for experimentally confirmed VISTA enhancers are similar (482/1,196  = 40.30% and 60/1,196  = 5.02% overlap A-Es and A-Ps, respectively), which suggests that our predicted active enhancers have real enhancer functions. A proportion of the VISTA enhancers not overlapping our predictions could be active specifically during development or in other cell types than our focus cell lines.

### DECRES extends the FANTOM enhancer atlas

Due to the limited depth of CAGE signals for eRNAs, a portion of active (or transcribed) enhancers will not have been detected in the original compilation of the enhancer atlas. Hence, we sought to identify additional partially supported enhancers for which eRNA signals were below the original atlas threshold settings [4]. In the previous work, a total of 200,171 bidirectionally transcribed (BDT) loci were detected across the human genome, using CAGE tags of 808 cell types and tissues. After excluding BDT loci within exons, a partially supported set of 102,021 BDT regions remained, of which 43,011 balanced loci (similar eRNA levels on both sides) constitute the FANTOM enhancer atlas [4]. In order to investigate whether more active enhancer candidates can be detected for each of the six cell types, we trained a MLP on its active atlas regions, and predicted classes for all 102,021 BDT sites. Among the 102,021 BDT loci, most were classified as negative regions in a given cell (Additional file 1: Table S5), while on average 13,316 were predicted as A-Es and only 834 were predicted as A-Ps per cell type. A substantial number (6535 on average) of inactive enhancers in the original enhancer atlas were predicted as active by our model (Additional file 1: Table S6), consistent with the assumption that BDT data is incomplete for any given sample. On average 5514 BDT loci excluded by the original atlas, were predicted as A-Es per cell type. Over the six analyzed cell types, a total of 38,601 BDT loci were predicted as A-Es (Additional file 3), of which 16,988 represent an expansion of the original FANTOM enhancer atlas. Note that 21,398 out of 43,011 enhancers from the original FANTOM enhancer atlas are not predicted as active in the six cells analyzed here, but these regions may be active in the other 802 cells for which there are inadequate features to analyze.

### Computational validation of DECRES’s prediction using functional and motif enrichment analysis

We performed functional enrichment analysis on the genome-wide predicted A-Es and A-Ps using GREAT [44]. For GM12878 cells, 79% of predicted enhancer regions are more than 5 kilobase pairs (kbps) away from gene TSSs (Additional file 1: Figure S15A), while 47% of predicted promoters are less than 5 kbps to the annotated gene TSSs (Additional file 1: Figure S15B). Similar statistics were obtained for the remaining five cell types. Annotation analyses of the GM12878-specific CRRs show that proximal genes are associated to: immune response from gene ontology (GO) annotations (Additional file 1: Figure S15C); B cell signalling pathways from MSigDB Pathway annotations (Additional file 1: Figure S15D); and leukemia from disease ontology annotations (Additional file 1: Figure S15E). Results are consistent with the lymphoblastoid lineage of the cells. Next, we performed functional enrichment analysis on the BDT-supported predicted enhancers not previously reported in the FANTOM enhancer atlas (“not in atlas”). Results are fully consistent with the above analysis (Additional file 1: Figure S16).

We further carried out motif enrichment analysis on the predicted cell-specific CRRs and not-in-atlas enhancers using HOMER [45]. The predicted regions are enriched for motifs similar to JASPAR binding profiles [46] (Additional file 1: Figure S15F and Figures S16-S26) both associated to TFs maintaining general cell processes and TFs with selective roles in cell-related functions. For instance, motifs for Jun-, Fos-, and Ets-related factors were enriched in regions from all six cell types. These TFs regulate general cellular progresses such as differentiation, proliferation, or apoptosis [47, 48]. Cell-appropriate TF enrichments were observed for each cell (summarized in Additional file 1: Table S7). For example, RUNX1 and other Runt-related factors, which play crucial roles in haematopoiesis, are observed in GM12878 (Additional file 1: Figure S15F and Figure S16) [49]. C/EBP-related factors that regulate genes involved in immune and inflammatory responses are expressed in cervix (Additional file 1: Figures S17 and S18) [50]. HNF1A, HNF1B, FOXA1, FOXA2, HNF4A, and HNF4G factors regulate liver-specific genes (Additional file 1: Figures S19 and S20) [51, 52]. NFY factors cooperate with GATA1 to mediate erythroid-specific transcription in K562 (Additional file 1: Figures S25 and S26) [53].

We performed functional and enrichment analysis on the A-E and A-P predictions from the Combined method [12], and report the results in Additional file 1: Figures S27-S30. Most of the predicted promoters by the Combined method are distal to known gene TSSs, which is similar to enhancers. For instance on cell line GM12878, only 22% of the Combined promoters are located less than 5 kbp to the annotated gene TSSs, compared to 47% of the DECRES promoters. Moreover, functional analysis on the CRRs predicted by the Combined method returned much less or zero significant terms for GO biological process, MSigDB pathway, and disease ontology than the DECRES predictions. The motif analysis results of both methods are consistent.

## Discussion

Our study brings together a large collection of high-throughput data from global projects to allow for supervised annotation. One key challenge in such analysis is the depth of validation. In this report, validation is assessed using existing collections of reliable enhancers, including CAGE [4], and laboratory validated sets from CRE-seq [14], and, on a small-scale, transgenic mouse assays [43]), showing that the supervised approach nears 89% sensitivity. While we compare to multiple laboratory validated sets retrospectively, a prospective assessment would have broad value. In light of the recent advances in both big data analysis methods and genome-scale data generation, we believe it is opportune to launch a global prospective assessment, such as enabled within the DREAM Challenges program [54]. Such a test for annotation of *cis*-regulatory regions in the human genome would inspire the machine learning community to push the performance limit of supervised CRR-prediction methods, and would encourage laboratory biologists to accelerate cell type-specific data generation.

Enhancers and promoters have both common and distinct characteristics. In our cross-validations, we show that A-E and A-P are highly separable (Fig. 1a), while better performance can be obtained if A-E and A-P are treated as a single class (Additional file 1: Figure S1C). Both continuous (merging enhancers and promoters together) and distinct models (treating enhancers and promoters separately) have limitations. While a continuous model may overlook functional differences, a distinct model may overemphasize such differences. A potentially better prediction model might require two hierarchical steps. It could first distinguish CRRs from the background genome, then assign a continuous score to each candidate region indicating the likelihood of being an enhancer. Further clustering and subtyping may be necessary. It is worth mentioning that the CAGE-defined enhancers used in this study may introduce some bias towards capturing a specific class of enhancers which exhibit reasonably strong and detectable transcription. To further investigate the characteristics of enhancers and improve genome-wide prediction, enhancers detected by other techniques, such as GRO-seq, will need to be considered in the future.

Our predicted CRRs take a substantial but small portion of the non-coding regions, previously known as “junk DNA”. It may be because only six cell types are considered in this work. Nevertheless, we have already seen that the non-coding regions exhibit regulatory functionality. It would be interesting in the next phase to collect data from a large number of cell types and examine the coverage, which will unveil whether regulatory regions have an oasis pattern. It may also imply that certain fragments of the non-coding regions play other partially known (such as suppression, domain boundary, and development) and unknown roles.

As already advocated in our review [7], two other deep learning models might be well suited to improve annotations of non-coding regions. One method is convolutional neural networks (CNNs), which can take into account the topological properties of features. The other is bidirectional recurrent neural networks (RNNs), which can consider the information from adjacent regions (i.e. the context). Such an approach can be potentially applied to annotate regulatory domains or complexes where exons, introns, promoters, enhancers, silencers, and insulators form cohorts for specific functionalities. Bidirectional RNNs have a smoothing effect, making the predictions context-dependent. Development of CNN- and RNN-based models for prediction of enhancers using sequence information has just emerged [55]. We foresee more sophisticated deep learning models in the near future for comprehensive genome annotations. To prevent predictions from jumping between states, smoothing has been taken into consideration in a deep neural network combined with hidden Markov model [56, 57]. Combined with MLPs, CNNs, or RNNs, other newly published deep feature selection techniques, such as layer-wise relevance propagation [58] and class saliency extraction [59], might be useful to identify informative signal peaks for *cis*-regulatory elements of focus. Furthermore, transfer learning [60] and multi-task learning [37] techniques might be useful in the design of deep predictive models, particularly when the number of learning examples of one cell type is limited or a region allows several annotations. Assessing the impact of sequence variations in non-coding regions on gene expression and phenotypes is of high clinical interest [32, 61], which was one motivation for the GTEx project [62]. The current predictions using MLPs and future annotations using CNNs and RNNs can integrate sequence variations (captured in alignment of short sequence reads of ChIP-seq and other sequencing techniques) and RNA-seq gene expression data of a cell type of interest, so that the impact of genetic variations in non-coding regions can be prioritized.

## Conclusions

Using FANTOM data for training, we show that supervised deep learning methods are able to accurately predict active enhancers and promoters across the human genome. Models incorporating cell-specific data outperform models restricted to universal data (e.g. sequence), and highlight key experimental features that tend to be incorporated into predictive models when available. We explore the relative performance of 2- and 3-class models that either group or separate enhancers and promoters. Finally, we deliver a comprehensive collection of annotations, that label 6.8% of the genome as enhancers and 0.6% as promoters in one or more of six well-characterized cells.

Accurate annotation of regulatory regions across the human genome is essential for genome interpretation. With genome sequencing transitioning to a standard clinical test, the ability to move beyond the analysis of protein-coding alterations has the potential to expand clinical diagnostic capacity to explain observed genetic disorders. By demonstrating the suitability of supervised deep learning methods to label regulatory regions, we now enter into a new stage of genome annotation. In the next few years, we anticipate that characterization of regulatory properties in specific cell populations will accelerate, using both chromatin-based and sequencing-based methods. As demonstrated in this report, deep learning methods are well suited for the challenge of using the expanded data for reliable annotation of the genome.

We anticipate that the collection of regulatory region annotations provided in this study will have broad utility for genome interpretation, and that the demonstration of the sufficiency of training data and the utility of deep learning supervised methods for CRR prediction will move the discussion to a highly applied period of high-quality annotation. Understanding how CRRs interact and how they link to their target genes is the key to decipher the *cis*-regulatory mechanism. We expect that further development of integrative machine learning methods [63, 64] is crucial to reconstruct such a gene regulatory system.

## Methods

### Data

For the purpose of supervised analysis, we collected feature data from ENCODE [10] along with the transcriptionally active enhancers and promoters from eight matched cell types catalogued by the FANTOM effort [4, 24]. These cell types include A549, GM12878, HelaS3, HepG2, HMEC, HUVEC, K562, and MCF7. For each cell type, we defined seven classes of labelled regions, including A-E, I-E, A-P, I-P, A-X, I-X, and UK. The libraries of enhancers and promoters were downloaded from [65]. A-Es and I-Es were defined as FANTOM enhancers with TPM >0 (tags per million) and TPM =0, respectively. A-Ps and I-Ps were randomly selected FANTOM promoters with TPM >5 and TPM =0, respectively. A-X and I-X were defined based on exons’ transcription levels measured by RNA-seq [66]. An exon with peak-max greater than 400 (equal to 0) was defined as A-X (I-X). The UK regions were sampled from the genome regions excluding all FANTOM CAGE tags, all exons, and DNaseI open regions. The numbers of labelled regions used in this study are listed in Additional file 1: Table S1. For each cell type, we built a comprehensive feature set, integrating histone modification and TF binding ChIP-seq, DNase-seq, FAIRE-seq, and ChIA-PET data from the ENCODE project [66, 67].

These features characterize the activities of enhancers and promoters in cell-specific aspects. Additionally, CpG islands and phastCons evolutionary conservation scores were included, because it is well recognized that some regulatory regions are highly GC-rich and extremely conserved. For each labelled region, the mean value of feature signals fall within a bin centered at the region was taken as the feature value using bwtool [68]. We tried different bin sizes including 200, 500, 1,000, 2,000, and 4,000 bps. Since 200 bps worked the best in cross-validation tests, we used it throughout our analyses. The numbers of features used for each cell type and the numbers of common features between any pair of them are given in Additional file 1: Table S2. A combined list of features is provided in Additional file 4. Our labelled data are downloadable from [69].

### Deep learning for classification

Based on the Deep Learning Tutorials [70] and Theano [71], we implemented a deep learning package named DECRES (DEep learning for identifying Cis-Regulatory ElementS and other applications) which is available at [69]. We applied a supervised deep model – feedforward neural network (also known as multilayer perceptrons or MLP) for the detection of regulatory regions. For each experiment, we conducted a model search from no hidden layers upto three hidden layers with maximally 256, 128, and 64 units in the first, second, and third hidden layers. Initial learning rate, *l*_2_-regularization amount (to control model complexity), and momentum (to stabilize the optimization) were searched across ranges of values. The maximum number of allowed iterations was set to 1000. The initial learning rate could reduce gradually as the number of iterations increases. Using batch-size 100, stochastic gradient descent was employed to optimize the model parameters. Rectified linear unit (ReLU) activation function [72] was used. When evaluating the classification performance of various experiments, we randomly sampled a balanced training set with maximally 70% of examples and maximally 3000 examples in each class, the remaining data were further randomly sampled to generate a corresponding test set with proportion of A-E:A-P:A-X:I-E:I-P:I-X:UK =1:1:1:2:2:1:10 to mimic the true genome-wide background among the classes (Same ratio was used in the comparison with ChromHMM and Combined methods). A training set was further partitioned into a training subset for model learning and a validation subset for early termination. We scaled each feature in the training subset to [0,1], and applied the same estimated scaling factors to the validation subset and test set. Class-wise rate (CWR, i.e. averaged sensitivity and specificity for two classes, and averaged sensitivities for more than two classes), area under the receiver operating characteristic curve (auROC), and area under the precision-recall curve (auPRC) were calculated to measure the classification performance. The above procedure was repeated 100 times to determine the means and variations of CWRs, auROCs, and auPRCs. Random forest [73] was compared on the same training-test splits in our experiments. Before predicting regulatory regions in the whole genome, all labelled A-E samples and 3000 samples in each of the other classes of a cell type were used to train the network.

### Feature selection

Based on our newly devised deep feature selection (DFS) model [26], we designed the randomized DFS (RDFS), which is a deep extension of randomized LASSO [74], for stably selecting subsets of discriminative features.

Addressing the limitations of sparse linear models for feature selection, DFS is able to model the non-linearity of the features and select a single subset of features for multi-class data. The main idea of DFS is to add a one-to-one linear layer (named feature-selection layer) to the above described feedforward neural network. For the *i*-th input feature *x*_*i*_, the output of the feature-selection layer becomes *w*_*i*_*x*_*i*_. Thus, the parameter of this layer is a vector *w*. By shrinking *w*, some of its elements turn to zeros, such that the corresponding features do not contribute to the classification at all. The upper hidden layers of the model have the capability of modelling non-linear interactions in the data. The feature selection layer allows to select a single subset of features for multi-class problem.

The randomized DFS procedure is similar to random forest, both implementing the philosophy of the wisdom of crowds. In randomized DFS, DFS with perturbed feature-wise sparsity control parameters runs on a training subset which is a randomly sampled portion (usually $\frac {1}{2}$) from the entire training set, and selects the top *K* features based on feature weights. This random procedure is repeated, say 100, times to generate the empirical probability of each feature being selected. The empirical probabilities are used as feature importance. In our experiment, we used ReLUs as activation functions and set *K*=10 in this procedure. We scaled each feature to [0,1] in the training set, and applied the estimated scaling factors from the training set to the test set. From the most important to the least important features, we kept adding features to a MLP to evaluate how many features are sufficient for the prediction. Randomized DFS was compared to random forest on the same partitions of training and test sets in this study. The DFS and randomized DFS models were included in the DECRES package.

Aiming at trading off the size of feature subset and classification performance (auPRC), we designed a method based on curving fitting that was applied in Fig. 4. Denoting a size of feature subset and corresponding test auPRC by *x* and *y* respectively, we first fit function $y=\frac {2s}{\pi }\arctan (kx)$ where *k* and *s* are scale parameters. Once done, a point can be chosen on the curve given a proper tangent value (say *t*) using $x_{t}=\frac {1}{k}\sqrt {\frac {2ks-t\pi }{t\pi }}$ and $y_{t}=\frac {2s}{\pi }\arctan (kx_{t})$. Since the values come from different scales on *x* and *y* axes, we qualitatively used *t*=0.5.

## Additional files

Additional file 1 This file contains supplemental **Tables S1-S7**, and supplemental **Figures S1-S30**. (PDF 27600 kb)

Additional file 2 Genome-wide predictions of *cis*-regulatory regions for all six cell types. (ZIP 20400 kb)

Additional file 3 Predictions on CAGE supported bidirectional loci. AiA: Active in the FANTOM Enhancer Atlas; IiA: Inactive in the FANTOM Enhancer Atlas; NiA: Not included in the FANTOM Enhancer Atlas; Specific: Predicted cell-specific A-Es. (ZIP 4820 kb)

Additional file 4 List of features used for each cell type. (ZIP 1.36 kb)

## Abbreviations

A-E: Active enhancer
A-P: Active promoter
A-X: Active exon
BDT: Bidirectionally transcribed
BG: Class merging I-E, I-P, A-X, I-X, and UK
auPRC: Area under the precision-recall curve
auROC: Area under the receiver operating characteristic curve
CAGE: Cap analysis of gene expression
ChIP-seq: Chromatin immunoprecipitation followed by sequencing
CNN: Convolutional neural network
CRR: *cis*-regulatory region
CWR: Class-wise rate, i.e. averaged sensitivity and specificity for two classes, and averaged sensitivities for more than two classes
DECRES: Deep learning for identifying *cis*-regulatory elements and other applications
DFS: Deep feature selection
DRM: Dinucleotide repeat motif
eRNA: Enhancer RNA
ENCODE: Encyclopedia of DNA Elements
FANTOM: Functional Annotation of the Mammalian Genome
GRO-seq: Global run-on and sequencing
I-E: Inactive enhancer
I-P: Inactive promoter
I-X: Inactive exon
MLP: Multilayer perceptron
MPRA: Massively parallel reporter assay
Pol2: RNA polymerase II
RDFS: Randomized deep feature selection
RF: Random forest
RNN: Recurrent neural network
ReLU: Rectified linear unit
TF: Transcription factor
TPM: tags per million
UK: Unknown/uncharacterized
