# Supplementary material for: Genome-wide prediction of cis-regulatory regions using supervised deep learning methods
Source: BMC Bioinformatics. 2018 May 31;19:202. doi: 10.1186/s12859-018-2187-1 (PMC5984344; doi:10.1186/s12859-018-2187-1)
Supplement: Supplementary file 1 — This file contains supplemental Tables S1-S7, and supplemental Figures S1-S30. (PDF 27600 kb) [file 12859_2018_2187_MOESM1_ESM.pdf]

Supplementary Tables and Figures for "Genome-Wide Prediction of cis-Regulatory Regions Using Supervised Deep Learning Methods"

Yifeng Li*[1,2], Wenqiang Shi†[1], and Wyeth W. Wasserman‡[1]

[1]Centre for Molecular Medicine and Therapeutics, BC Children's Hospital Research Institute, Department of Medical Genetics, University of British Columbia
[2]Digital Technologies Research Centre, National Research Council Canada

Table S1: Numbers of labelled regions in our data.

Cell	A-E	I-E	A-P	I-P	A-X	I-X	UK	Total
A549	387	40,387	10,907	128,998	8,998	14,712	81,217	285,606
GM12878	2,878	28,156	10,816	73,891	8,226	19,078	80,004	223,049
HelaS3	1847	32,179	10,759	79,009	9,123	22,071	81,502	236,485
HepG2	1465	34,556	11,467	96,184	9,931	19,071	79,417	252,091
HUVEC	1226	35,143	11,254	101,861	9,739	18,249	80,333	257,805
K562	894	34,392	10,076	82,829	9,033	20,261	78,081	235,566
MCF7	249	36,873	10,733	81,510	10,829	13,653	82,663	236,510

Table S2: Number of features used for each cell type and number of common features between any two cell types in our labelled data.

Cell	A549	GM12878	HelaS3	HepG2	HMEC	HUVEC	K562	MCF7
A549	45	38	26	38	15	19	38	25
GM12878	-	101	50	54	16	21	69	26
HelaS3	-	-	74	43	16	22	56	23
HepG2	-	-	-	72	16	21	57	28
HMEC	-	-	-	-	16	16	16	9
HUVEC	-	-	-	-	-	24	24	13
K562	-	-	-	-	-	-	135	29
MCF7	-	-	-	-	-	-	-	38

*E-mail address: yifeng@cmmt.ubc.ca, and yifeng.li@nrc-cnrc.gc.ca.
†E-mail address: shi@cmmt.ubc.ca.
‡Corresponding author, E-mail address: wyeth@cmmt.ubc.ca.

Table S3: Confusion matrices of classifying CRE-seq and MPRA validated regions using DECRES.

Original Class\DECRES Class	A-E	A-P	BG	Total
CRE-seq Positive Combined Predicted Enhancer in K562	254	0	132	386
CRE-seq Negative Combined Predicted Enhancer in K562	433	1	378	812
Combined Predicted Repressed Region in K562	0	0	298	298
Total	687	1	808	1496
MPRA Positive Enhancer in K562	120	0	2	122
MPRA Negative Enhancer in K562	179	0	15	194
Total	299	0	17	316
MPRA Positive Enhancer in HepG2	182	1	3	186
MPRA Negative Enhancer in HepG2	217	5	45	267
Total	399	6	48	453

Table S4: Numbers of predicted and cell-specific *cis*-regulatory regions in the whole human genome. Columns 2-3: results of two-class prediction. Columns 4-7: results of three class prediction.

Cell	A-E+A-P	Specific	A-E	Specific	A-P	Specific
GM12878	90,192	27,904	70,905	27,185	19,287	6,090
HelaS3	100,102	22,268	92,509	22,866	7,593	979
HepG2	114,873	35,986	105,007	41,109	9,866	2,135
HMEC	104,621	28,774	88,803	26,262	15,818	3,043
HUVEC	110,347	16,415	97,069	35,073	13,278	582
K562	133,940	30,835	122,321	56,374	11,619	371

Table S5: Numbers of predicted A-Es and A-Ps on the 102,021 BDT loci. AiA: Active in the FANTOM Enhancer Atlas. IiA: Inactive in the FANTOM Enhancer Atlas. NiA: Not included in the FANTOM Enhancer Atlas. Specific: Predicted cell-specific A-Es.

Cell	A-E	A-P	BG
GM12878	11,910	1,975	88,136
HelaS3	12,743	226	89,052
HepG2	10,761	488	90,772
HMEC	13,356	1,267	87,398
HUVEC	17,192	758	84,071
K562	13,936	288	87,797

Table S6: Overlap between our predicted A-Es and the FANTOM enhancer atlas.

Cell\Element	AiA	IiA	NiA	Specific
GM12878	2,088	5,144	4,678	4,199
HelaS3	1,459	6,435	4,849	1,073
HepG2	1,178	4,888	4,695	1,495
HMEC	1,250	6,644	5,462	1,916
HUVEC	879	8,716	7,597	3,560
K562	750	7,383	5,803	2,965

Table S7: Transcription factors whose binding motifs are enriched in specific cells.

Cell	Transcription Factor	Functionality
GM12878	RUNX1	RUNX1 and other Runt-related factors play crucial roles in haematopoiesis. Translocation of RUNX1 leads to severe acute myeloid leukemia [11].
GM12878	REL/NF-κB Factors	Play a critical role in immune response to infection [4].
HelaS3	C/EBP-Related Factors	C/EBP-related factors, a subfamily of the basic leucine zipper factors (bZIP), regulating genes involved in immune and inflammatory responses, are expressed in cervix [1].
HelaS3	CREB-Related Factors (Another bZIP Subfamily)	Possibly up-regulate Bcl-2 expression in apoptotic HeLa cells induced by trichosanthin [15].
HelaS3	TEAD1	Plays a role of apoptotic resistance in Hela cells [9].
HelaS3	AP-2 Factors	Can act as tumor suppressor, and malfunction of AP-2 was found in cervical cancer cells [2].
HelaS3	HOX Factors	a subgroup of HOX genes involve in cervical carcinoma [8].
HepG2	HNF1A, HNF1B, FOXA1, FOXA2, HNF4A, and HNF4G	These factors are hepatocyte nuclear factors that regulate liver-specific genes [3, 16].
HepG2	C/EBPs	Have a pivotal role in liver development and function [13].
HMEC	TEAD Family	Members of this family regulate epithelial-mesenchymal transition [17].
HUVEC	GATA Factors	Involve in networks of key determinants of vascular endothelial cell identity [7].
HUVEC	SOX Factors	Regulate vascular cell development and growth (vasculogenesis) [12].
HUVEC	CREB-Related Factors	Involve in several specific pathways in HUVEC cell [14].
K562	GATA-Type, NFYA, and NFYB USF1	The NFY factors cooperate with GATA1 to mediate erythroid-specific transcription, and coassociate with FOS binding to both promoters and enhancers in K562 [5]. The NFY factors are also found cooperate with USF1 and USF2 to active HOXB4 for hematopoiesis [18].
K562	STAT5	Maintains the high-level cell proliferation of K562 [10].

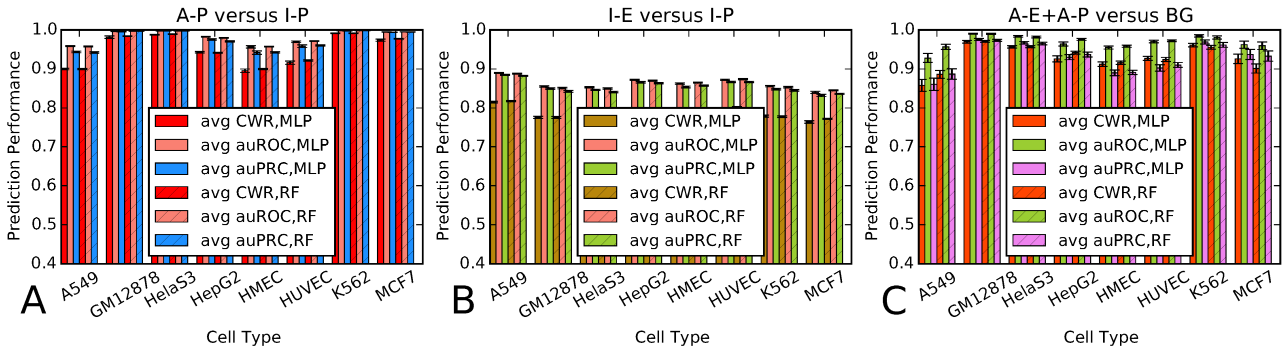

Figure S1: Mean performance and standard deviation of 10-fold cross-validations using the MLP model on our labelled data of eight cell types. A-E: Active Enhancer, A-P: Active Promoter, A-X: Active Exon, I-E: Inactive Enhancer, I-P: Inactive Promoter, I-X: Inactive Exon, UK: Unknown or Uncharacterized, BG: I-E+I-P+A-X+I-X+UK.

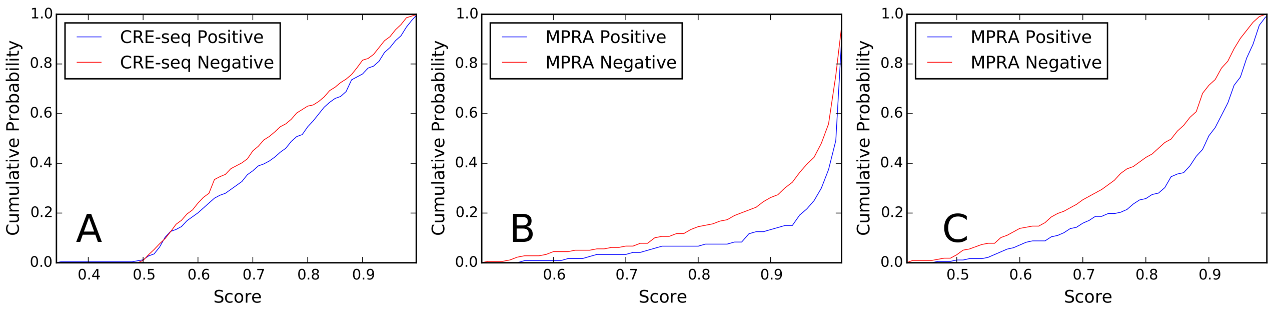

Figure S2: Cumulative DECRES membership probabilities (scores) of enhancers that were tested as positives and negatives by CRE-seq or MPRA. These enhancers were predicted as A-Es by DECRES (See Table S3).

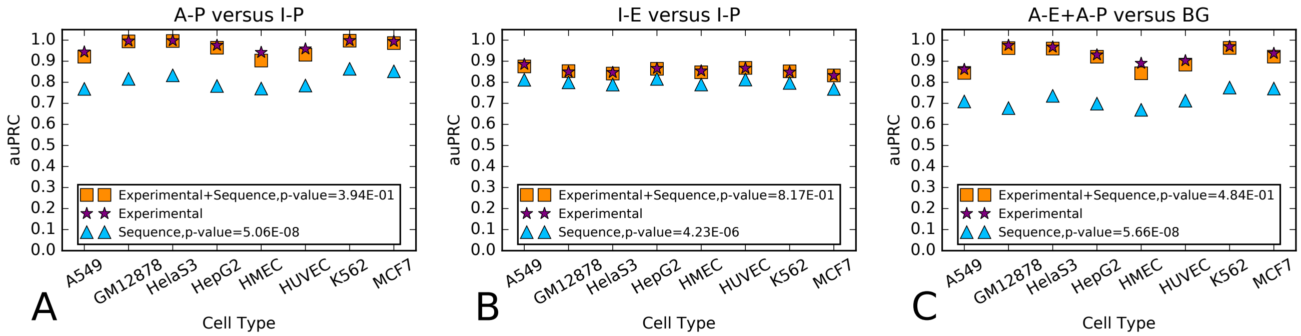

Figure S3: Comparing the mean auPRCs over 100 resampling and retraining on our labelled regions using different feature sets. "Experimental" means our experimentally derived next generation sequencing feature set. "Sequence" means the set of 351 sequence properties used in [6]. "Experimental+Sequence" means the combination of these two sets. The p-values in each legend were obtained using two-tailed Student's t-test to compare "Experimental"-based results with "Experimental+Sequence"-based and "Sequence"-based results, respectively.

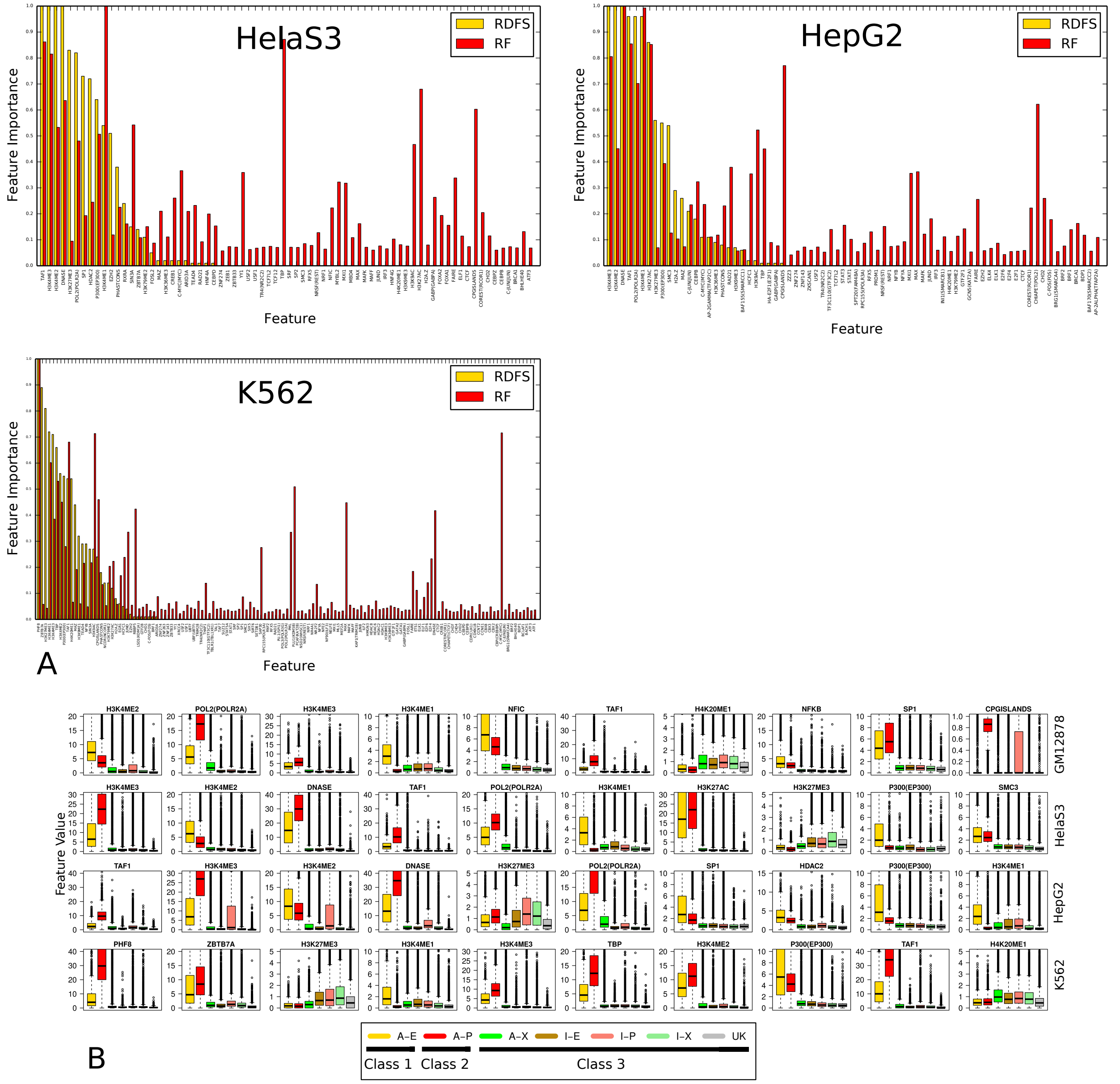

Figure S4: Feature importance and box plots of top features in the 3-class (A-E versus A-P versus BG) scenario. A: Feature importance discovered by randomized DFS (RDFS) and random forest (RF) on HelaS3, HepG2 and K562 cells. RF's feature importance scores were normalized to [0,1] for better comparison with RDFS. B: For the top 10 features of the 3-class models generated for four well-characterized cell lines, box plots depict the range of observed feature values (log2 scale) for 7 sequence classes.

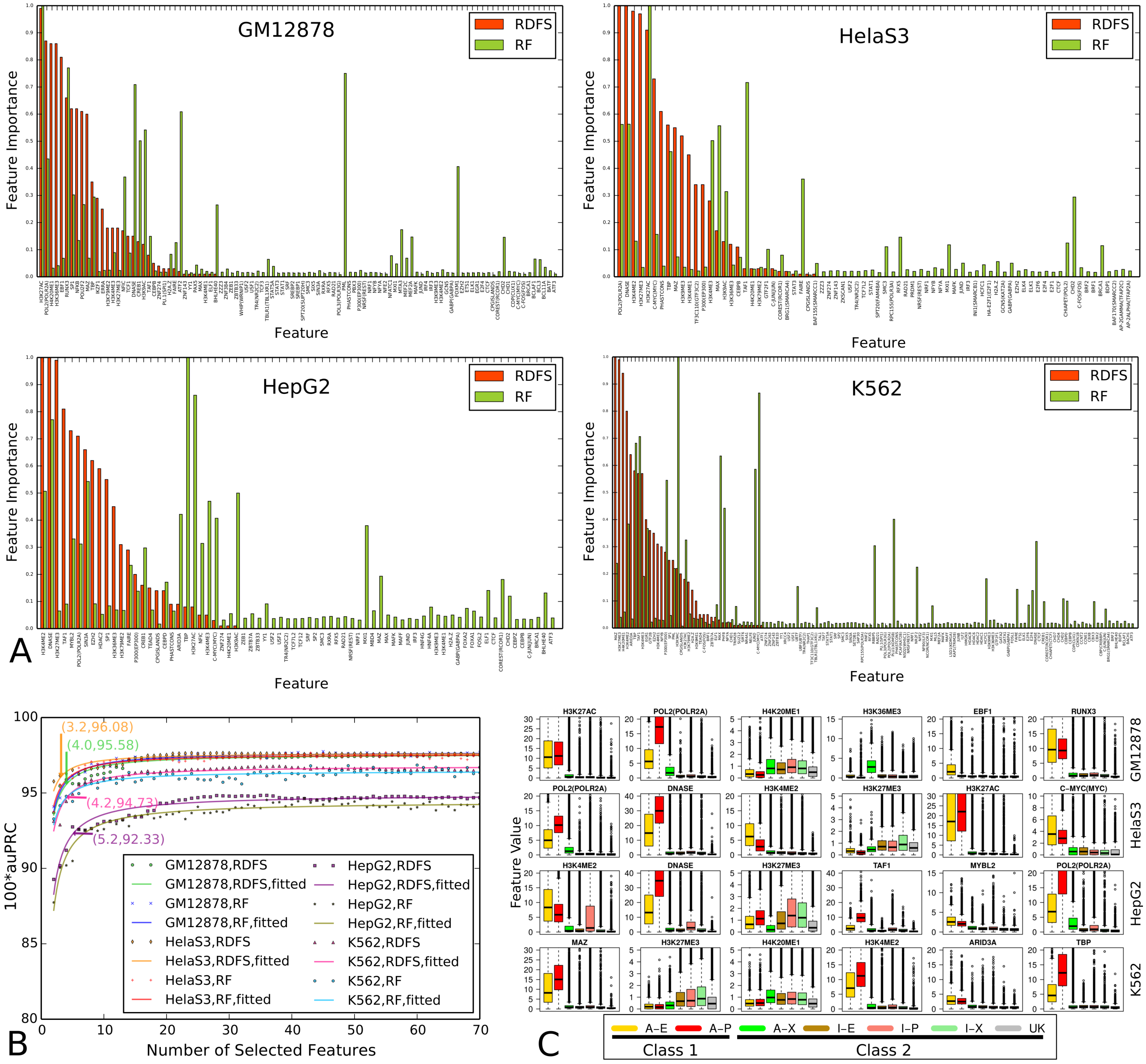

Figure S5: Feature importance, classification performance, and top features in the 2-class (A-E+A-P versus BG) scenario. A: Feature importance discovered by randomized DFS (RDFS) and random forest (RF). The random forest's feature importance scores were normalized to [0,1] for better comparison with randomized DFS. B: auPRC versus the number of features incorporated into the RDFS and RF. The annotated points indicate where a line with slope 0.5 intersects a fitted curve). C: For the top 6 features of the 2-class models generated for four well-characterized cell lines, box plots depict the range of observed feature values (log2 scale) for 7 sequence classes.

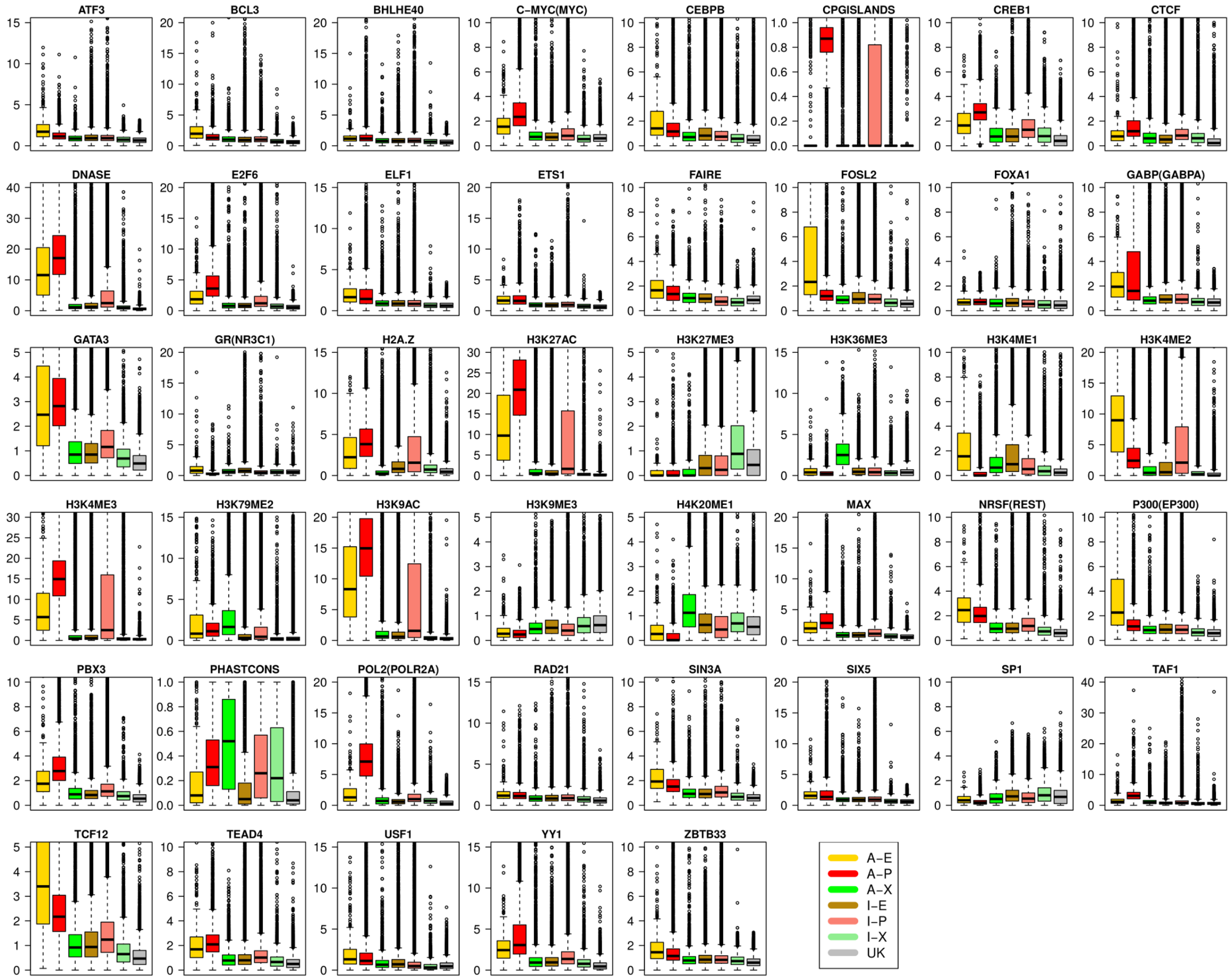

Figure S6: Box plots of all features for A549.

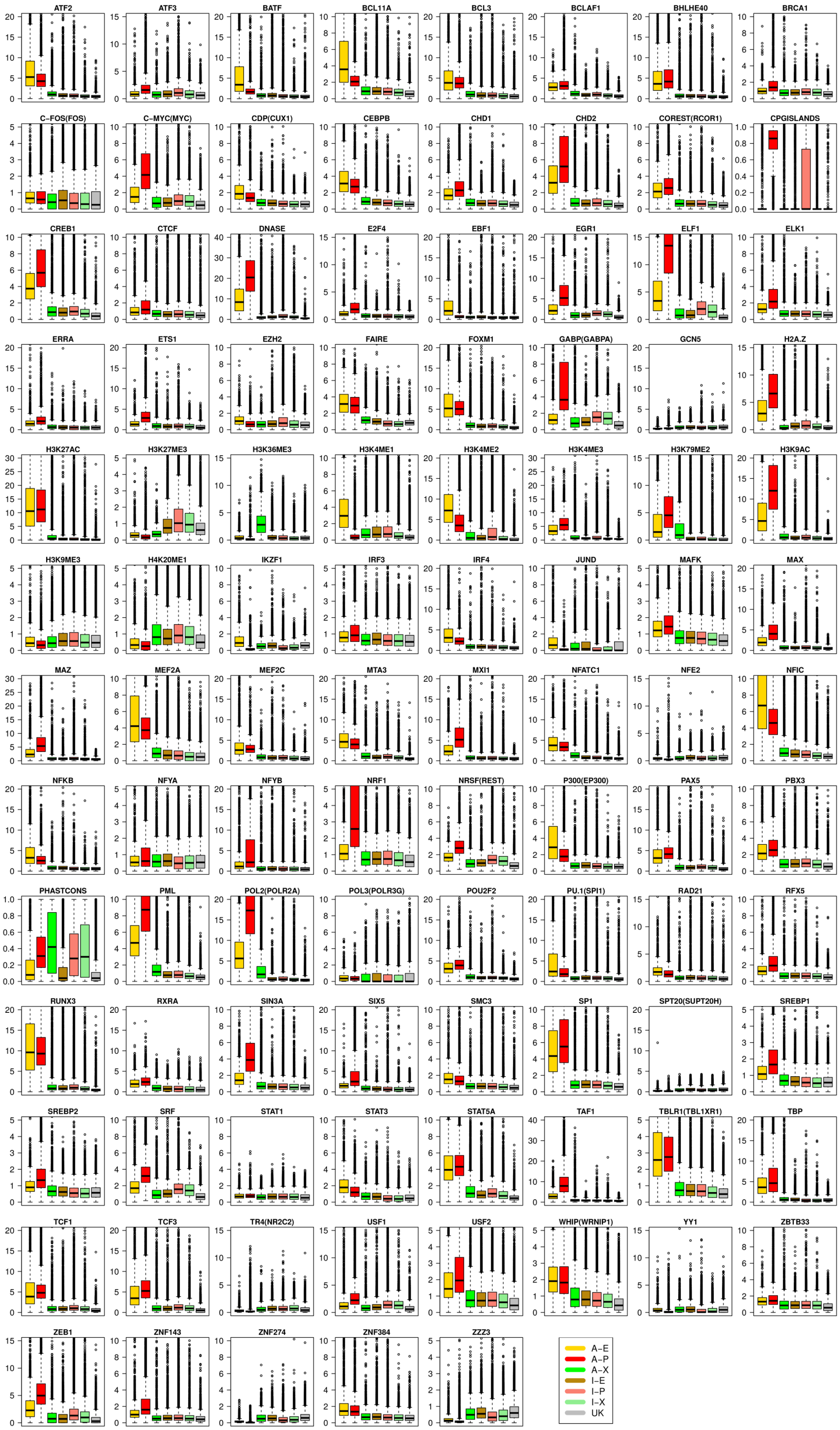

Figure S7: Box plots of all features for GM12878.

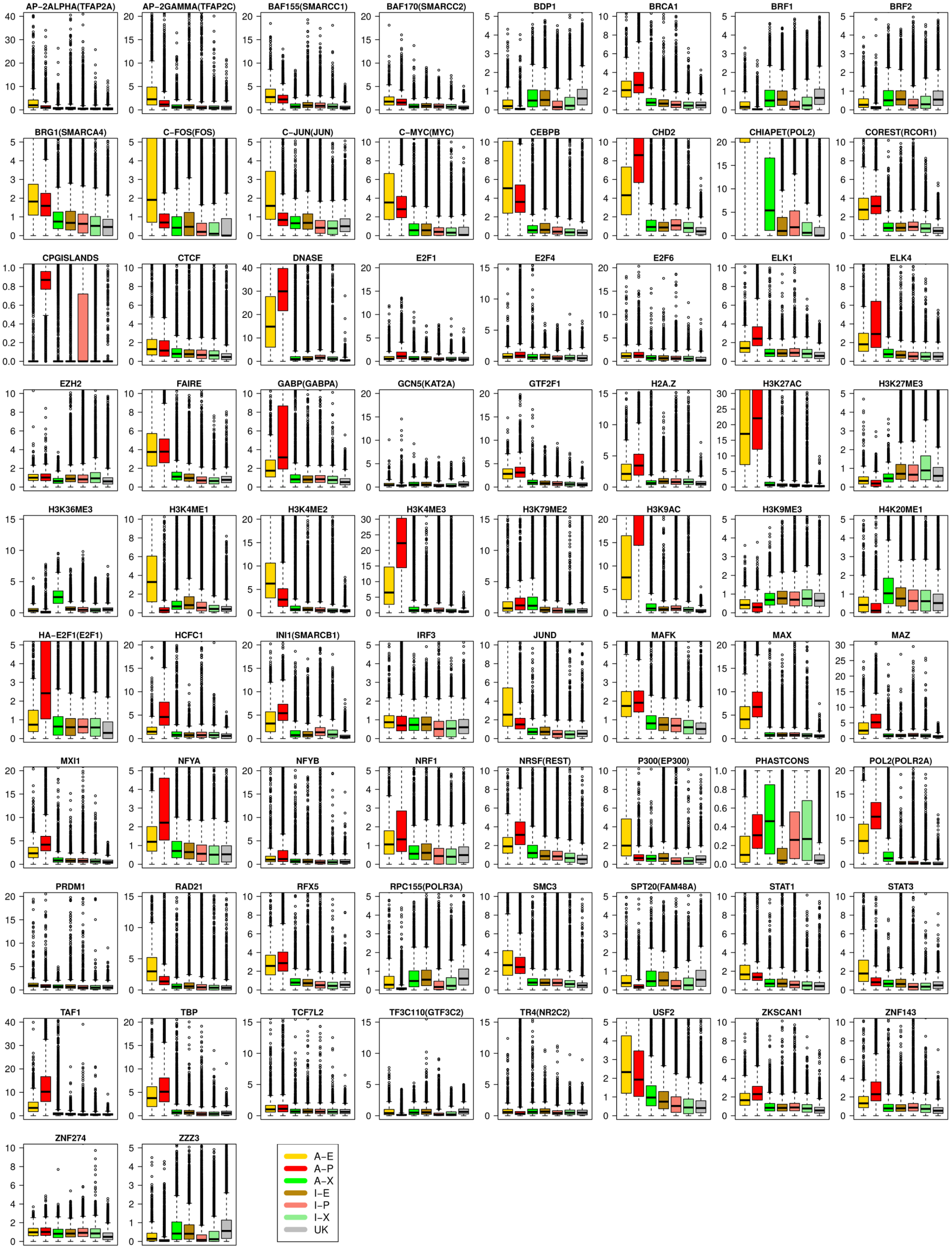

Figure S8: Box plots of all features for HelaS3.

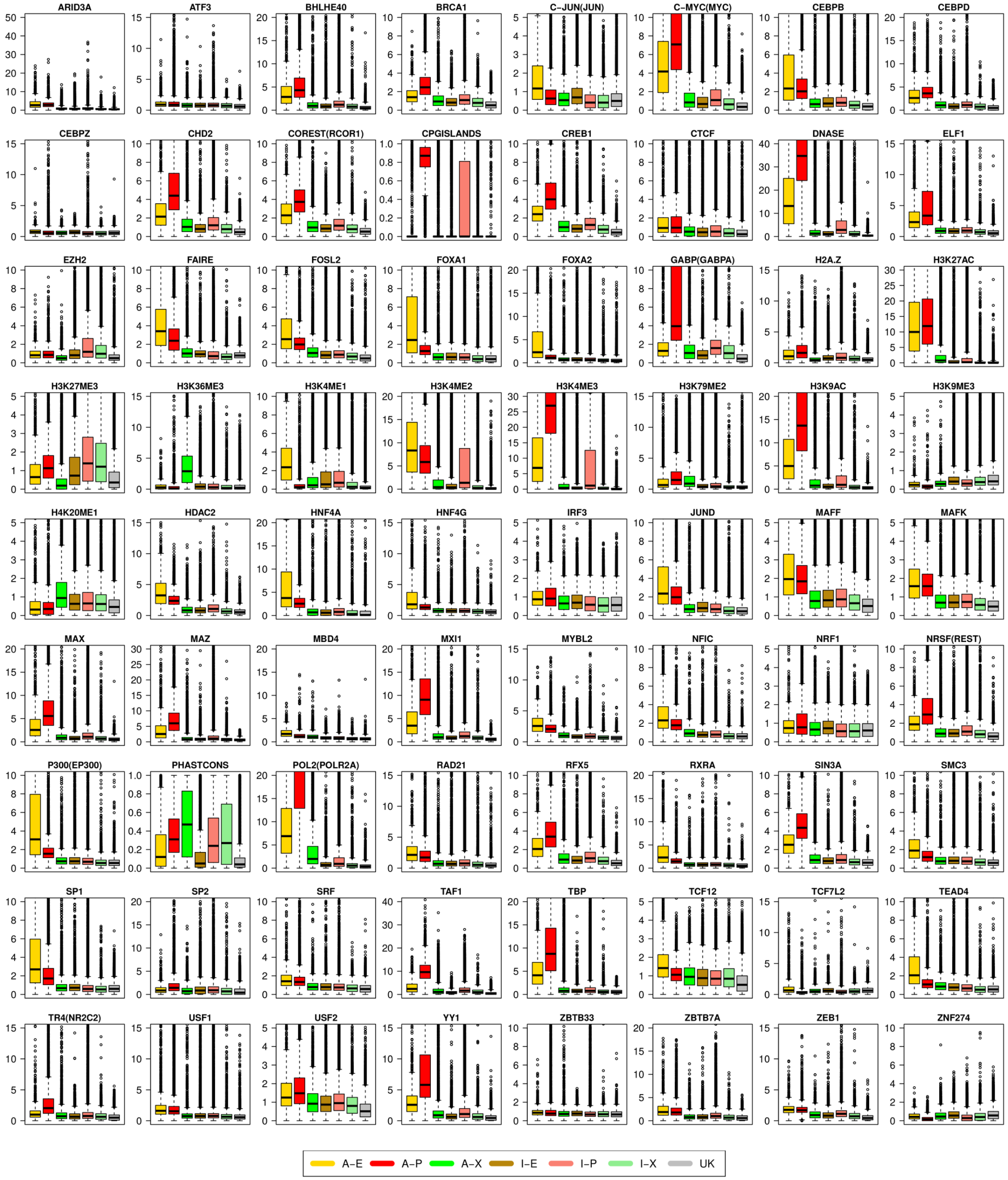

Figure S9: Box plots of all features for HepG2.

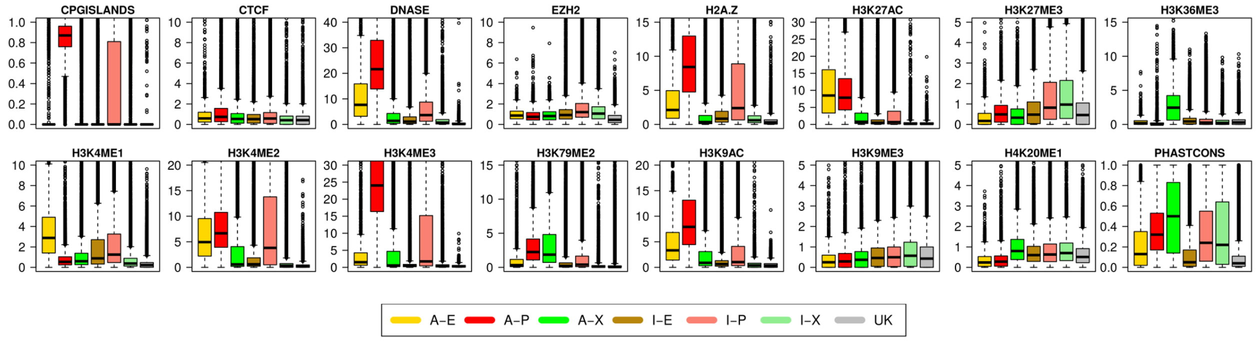

Figure S10: Box plots of all features for HMEC.

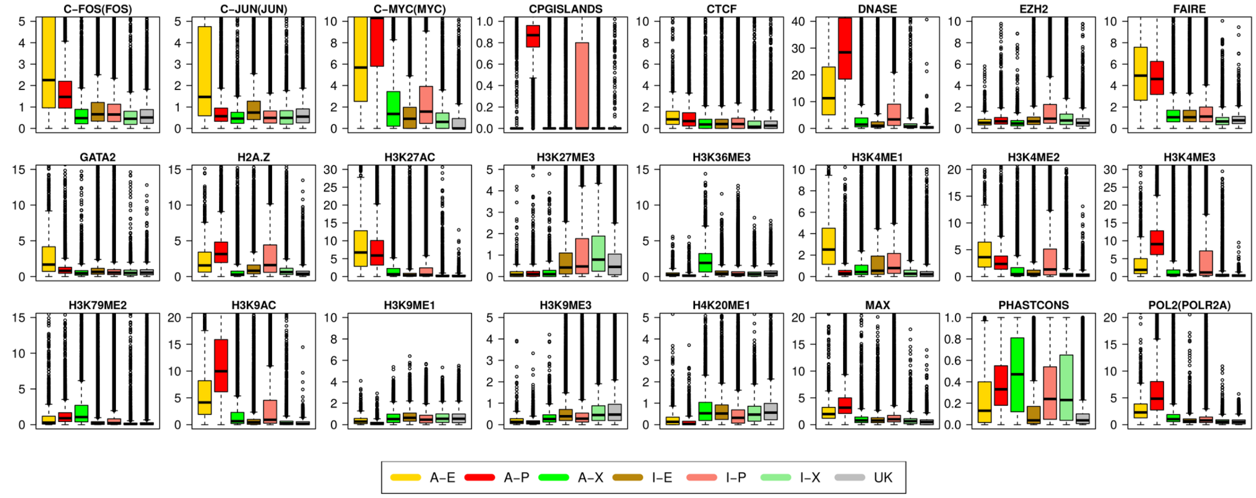

Figure S11: Box plots of all features for HUVEC.

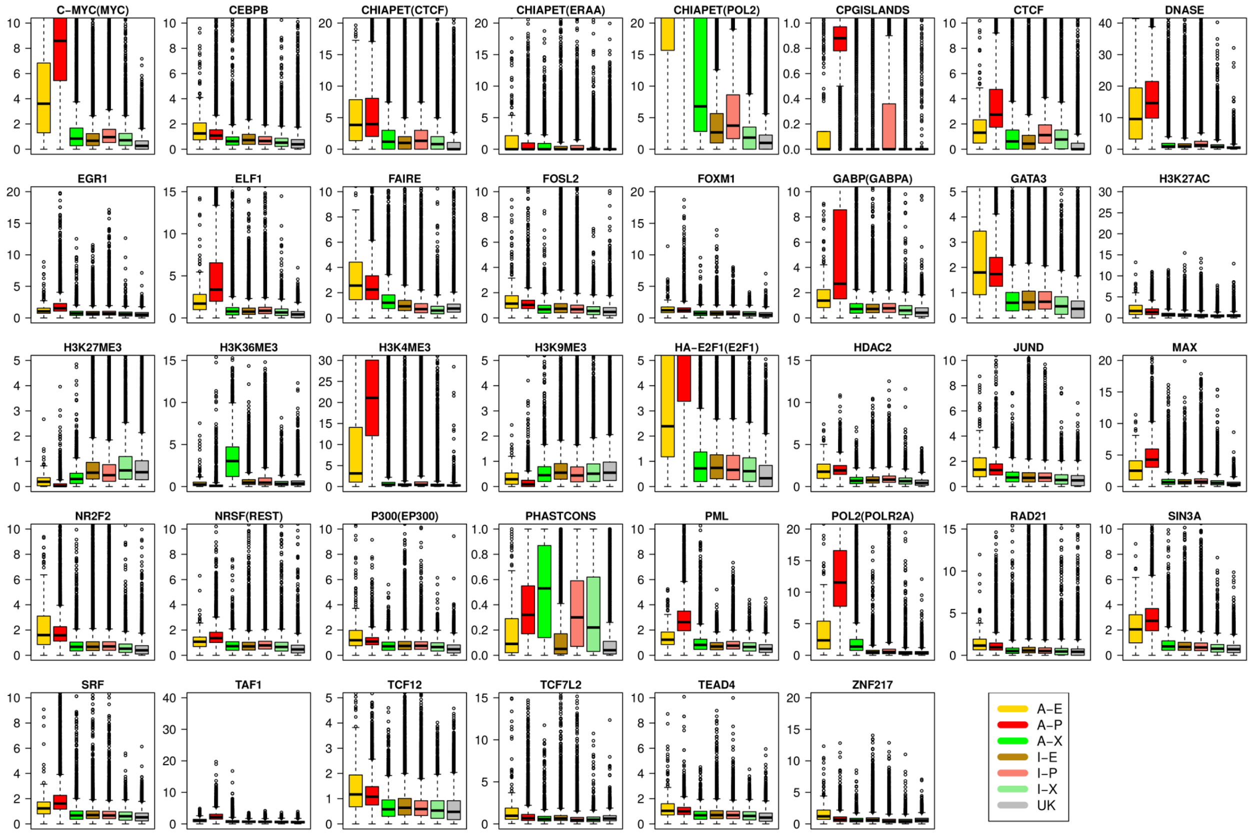

Figure S12: Box plots of all features for MCF7.

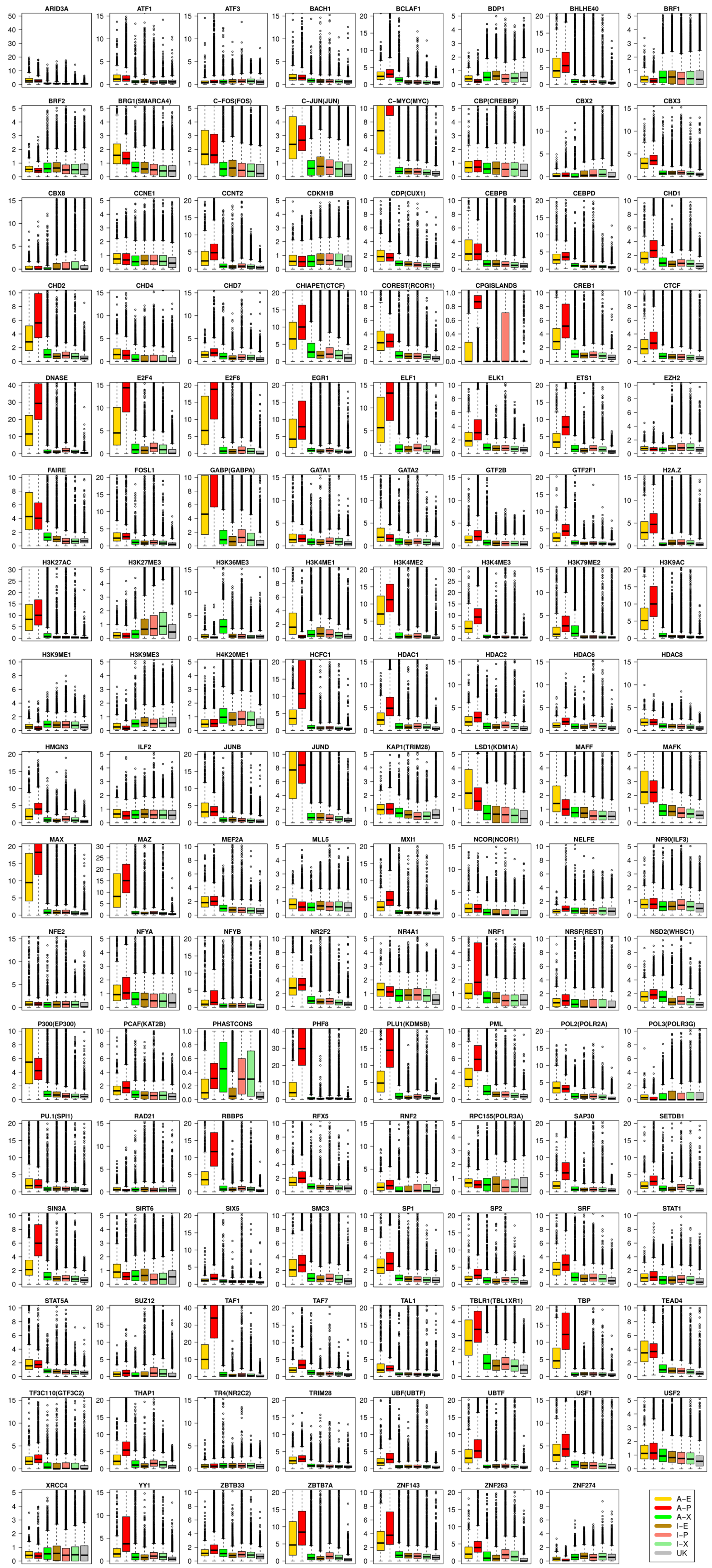

Figure S13: Box plots of all features for K562.

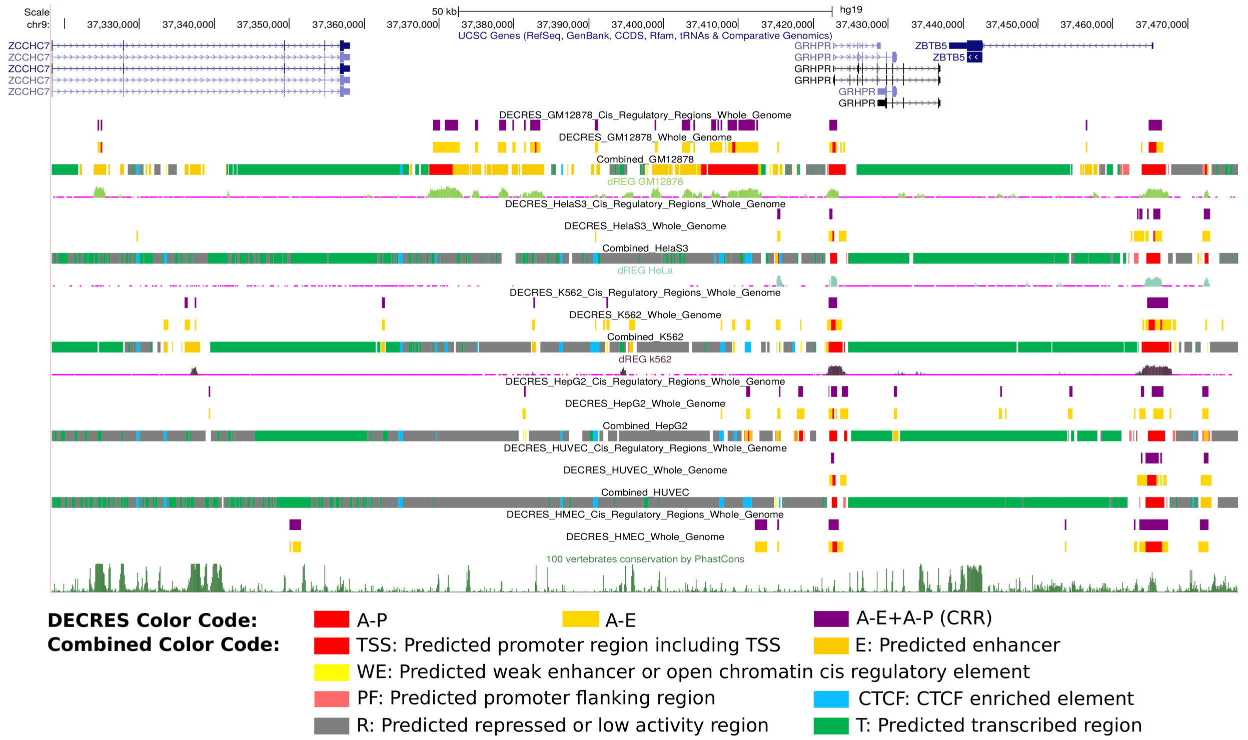

Figure S14: An example of predicted regulatory regions in the UCSC Genome Browser.

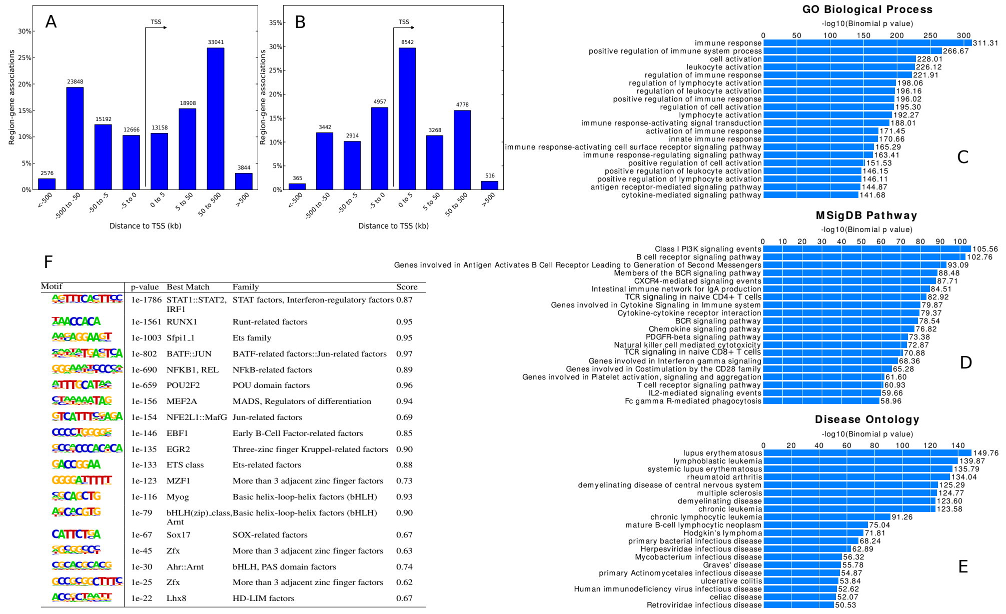

Motif	p-value	Best Match	Family	Score
	1e-1786	STAT1::STAT2, IRF1	STAT factors, Interferon-regulatory factors	0.87
	1e-1561	RUNX1	Runt-related factors	0.95
	1e-1003	Sfpi1_1	Ets family	0.95
	1e-802	BATF::JUN	BATF-related factors::Jun-related factors	0.97
	1e-690	NFKB1, REL	NFkB-related factors	0.89
	1e-659	POU2F2	POU domain factors	0.96
	1e-156	MEF2A	MADS, Regulators of differentiation	0.94
	1e-154	NFE2L1::MafG	Jun-related factors	0.69
	1e-146	EBF1	Early B-Cell Factor-related factors	0.85
	1e-135	EGR2	Three-zinc finger Kruppel-related factors	0.90
	1e-133	ETS class	Ets-related factors	0.88
	1e-123	MZF1	More than 3 adjacent zinc finger factors	0.73
	1e-116	Myog	Basic helix-loop-helix factors (bHLH)	0.93
	1e-79	bHLH(zip)_class, Arnt	Basic helix-loop-helix factors (bHLH)	0.90
	1e-67	Sox17	SOX-related factors	0.67
	1e-45	Zfx	More than 3 adjacent zinc finger factors	0.63
	1e-30	Ahr::Arnt	bHLH, PAS domain factors	0.74
	1e-25	Zfx	More than 3 adjacent zinc finger factors	0.62
	1e-22	Lhx8	HD-LIM factors	0.67

Figure S15: Functional and motif analysis of the DECRES genome-wide predictions on cell line GM12878. A: Distance from the predicted A-Es to gene TSSs. B: Distance from the predicted A-Ps to gene TSSs. C,D,E: Top 20 enriched biological processes, pathways, and diseases, respectively, in the predicted cell-specific CRRs. F: Enriched *de novo* motifs in the predicted cell specific CRRs. Column 4: families of best-matched TFs. Column 5: best match scores.

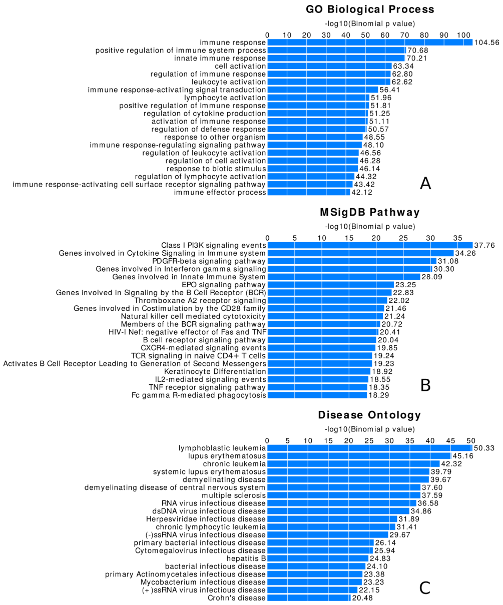

Motif	p-value	Best Match	Family	Score
	1e-150	FOS, JUN	FOS-related factors, Jun-related factors	0.96
	1e-136	Erg	Ets-related factors	0.93
	1e-104	REL	NFkB-related factors	0.96
	1e-95	RUNX1	Runt-related factors	0.95
	1e-66	Irf5	Interferon-regulatory factors	0.80
	1e-38	POU2F2	POU domain factors	0.93
	1e-38	SP1	Three-zinc finger Kruppel-related factors	0.85
	1e-29	Gm397_2	BetaBetaAlpha-zinc finger	0.72
	1e-28	DCE_S_III	Unknown	0.75
	1e-28	Gcm1_1	Glial cells missing (GCM)	0.72
	1e-24	Pax2	Paired domain only	0.75
	1e-24	Bhlhb2_1	Helix-Loop-Helix	0.86
	1e-22	Srf_2	MADS	0.75
	1e-20	KLF5	Three-zinc finger Kruppel-related factors	0.71
	1e-20	NFYB	Heteromeric CCAAT-binding factors	0.96
	1e-18	ETS::E-box	ETS::bHLH	0.65
	1e-18	USF2	bHLH-ZIP factors	0.68
	1e-16	MEF2A	MADS, Regulators of differentiation	0.86
	1e-15	CREB1	CREB-related factors	0.72
	1e-14	Foxo1	Forkhead box factors	0.69
	1e-14	Mycn	bHLH-ZIP factors	0.78
	1e-12	Hnf1b	POU domain factors	0.68
	1e-12	Irf6_2	Interferon-regulatory factors	0.61

D

Figure S16: Functional and motif analysis of the DECRES NiA enhancers on cell line GM12878. A,B,C: Top 20 enriched biological processes, pathways, and diseases, respectively, in the NiA enhancers. D: Enriched *de novo* motifs in the NiA enhancer regions. Column 4: the families of best-matched TFs. Column 5: best match scores.

Motif	p-value	Best Match	Family	Score
	1e-2223	CTCF	More than 3 adjacent zinc finger factors	0.90
	1e-2062	JUND, FOS	Jun-related factors, Fos-related factors	0.97
	1e-855	bZIP_cEBP-like_subclass	C/EBP-related factors	0.91
	1e-637	TFAP2A	AP-2	0.93
	1e-279	Klf1, Klf4	Three-zinc finger Kruppel-related factors	0.91
	1e-263	Nr2f2_1	Hormone-nuclear receptor	0.86
	1e-199	USF2, USF1	bHLH-ZIP factors	0.92
	1e-162	TEAD1	TEF-1-related factors	0.89
	1e-161	NFIC::TLX1	Nuclear factor 1::NK-related factors	0.81
	1e-135	Six1	Homeobox	0.91
	1e-87	GATA3	GATA-type zinc fingers	0.98
	1e-79	Tcfap2b_1	Helix-Loop-Helix	0.67
	1e-79	Foxo1	Forkhead box factors	0.75
	1e-77	Chop/Ddit3	C/EBP-related factors	0.93
	1e-67	Forkhead_class	Forkhead box factors	0.94
	1e-67	NFIC	Nuclear factor 1	0.86
	1e-51	Hoxa9	HOX-related factors	0.96
	1e-40	at_AC_acceptor	Unknown	0.61
	1e-33	GFY::Staf	Unknown::Zinc finger	0.79
	1e-30	Hoxc12	HOX-related factors	0.77

C

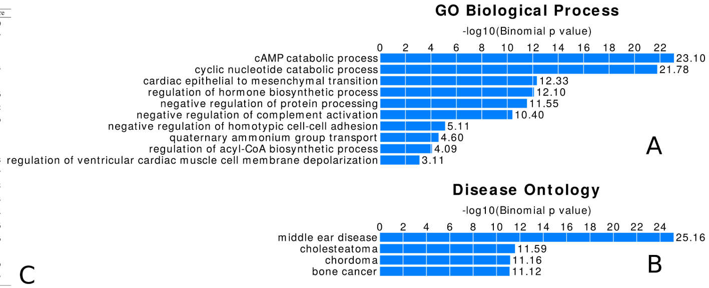

Figure S17: Functional and motif analysis of the DECRES genome-wide predictions on cell line HelaS3. A,B: Top enriched biological processes and diseases (no pathways enriched for HelaS3), respectively, in the predicted cell-specific CRRs. C: Enriched *de novo* motifs in the predicted cell specific CRRs. Column 4: families of best-matched TFs. Column 5: best match scores.

Motif	p-value	Best Match	Family	Score
	1e-413	FOS, JUN	Fos-related factors, Jun-related factors	0.97
	1e-79	KLF5	Three-zinc finger Kruppel-related factors	0.95
	1e-61	CEBPA	C/EBP-related factors	0.91
	1e-46	bZIP_CREB/G-box-like_subclass	CREB-related factors	0.84
	1e-45	FLI1	Ets-related factors	0.91
	1e-32	CTCF	More than 3 adjacent zinc finger factors	0.87
	1e-30	FOXD1	Forkhead box factors	0.82
	1e-30	Sp4_2	BetaBetaAlpha-zinc finger	0.82
	1e-26	REL	NFkB-related factors	0.92
	1e-24	SP1	Three-zinc finger Kruppel-related factors	0.73
	1e-24	Hoxc9	HOX-related factors	0.73
	1e-22	NFIC	Nuclear factor 1	0.78
	1e-22	Zbtb12_2	BetaBetaAlpha-zinc finger	0.64
	1e-20	TEAD1	TEF-1-related factors	0.79
	1e-19	Foxo1	Forkhead box factors	0.72
	1e-19	Hand1::Tcfe2a	Helix-Loop-Helix	0.62
	1e-18	ETS::E-box	Ets::bHLH	0.79
	1e-16	CEBPA	C/EBP-related factors	0.69
	1e-16	TBP	TBP-related factors	0.73
	1e-16	NFIC::TLX1	Nuclear factor 1::NK-related factors	0.65
	1e-16	Tcfap2e_2	Helix-Loop-Helix	0.76
	1e-15	Gata4	GATA-type zinc fingers	0.88
	1e-14	NFIC	Nuclear factor 1	0.60

D

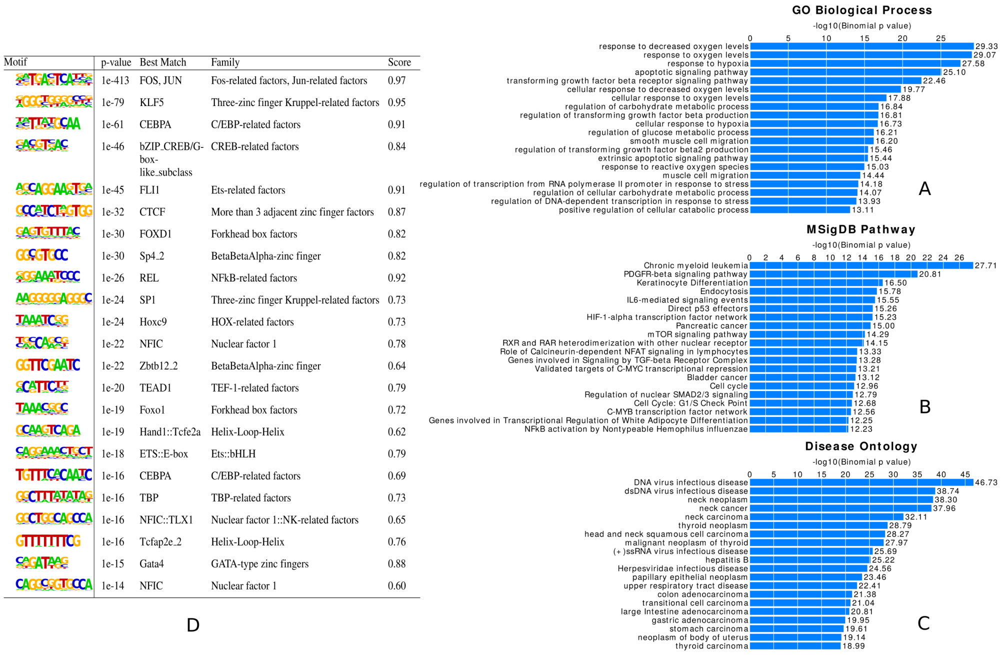

Figure S18: Functional and motif analysis of the DECRES NiA enhancers on cell line HelaS3. A,B,C: Top 20 enriched biological processes, pathways, and diseases, respectively, in the NiA enhancers. D: Enriched *de novo* motifs in the NiA enhancer regions. Column 4: families of best-matched TFs. Column 5: best match scores.

Motif	p-value	Best Match	Family	Score
	1e-3527	HNF4A, HNF4G	RXR-related receptors (NR2)	0.97
	1e-2261	FOXA1, Foxa2	Forkhead box factors	0.98
	1e-969	REST	Factors with multiple dispersed zinc fingers	0.82
	1e-571	REST	Factors with multiple dispersed zinc fingers	0.78
	1e-480	CTCF	More than 3 adjacent zinc finger factors	0.92
	1e-455	Esrrb	Steroid hormone receptors (NR3)	0.95
	1e-453	HNF1B, HNF1A	POU domain factors	0.89
	1e-366	bZIP_cEBP-like_subclass	C/EBP-related factors	0.92
	1e-337	Arid5a_1	ARID-related factors	0.67
	1e-283	Nuclear _Receptor _class	Unknown	0.78
	1e-206	TEAD1	TEF-1-related factors	0.90
	1e-161	TCF7L2	TCF-7-related factors	0.91
	1e-144	Tcf7l2_2	TCF-7-related factors	0.76
	1e-110	ELF1	Ets-related factors	0.92
	1e-108	JUND, FOS	Jun-related factors, Fos-related factors	0.97
	1e-78	Zfp691_1	BetaBetaAlpha-zinc finger	0.60
	1e-74	SP1	Three-zinc finger Kruppel-related factors	0.92
	1e-67	Smad3_1	SMAD factors	0.55
	1e-65	Zfp161_2	BetaBetaAlpha-zinc finger	0.71
	1e-61	Atoh1	Tal-related factors	0.75
	1e-55	Nkx2-5(var.2)	NK-related factors	0.59
	1e-46	DCE_S_II	Unknown	0.63

D

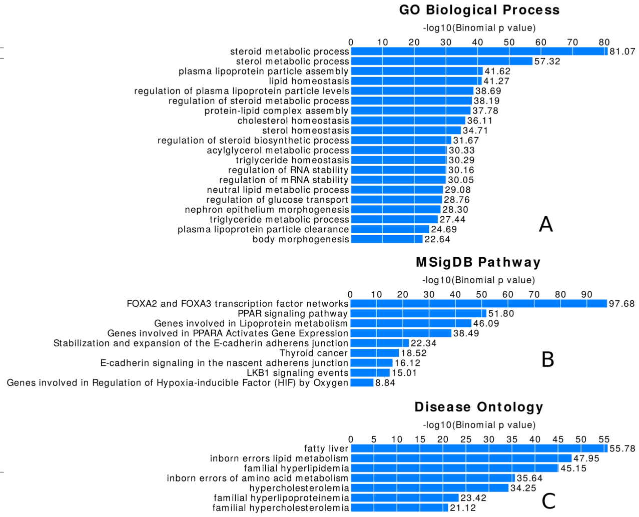

Figure S19: Functional and motif analysis of the DECRES genome-wide predictions on cell line HepG2. A,B,C: Top enriched biological processes,pathways and diseases, respectively, in the predicted cell-specific CRRs. D: Enriched *de novo* motifs in the predicted cell specific CRRs. Column 4: families of best-matched TFs. Column 5: best match scores.

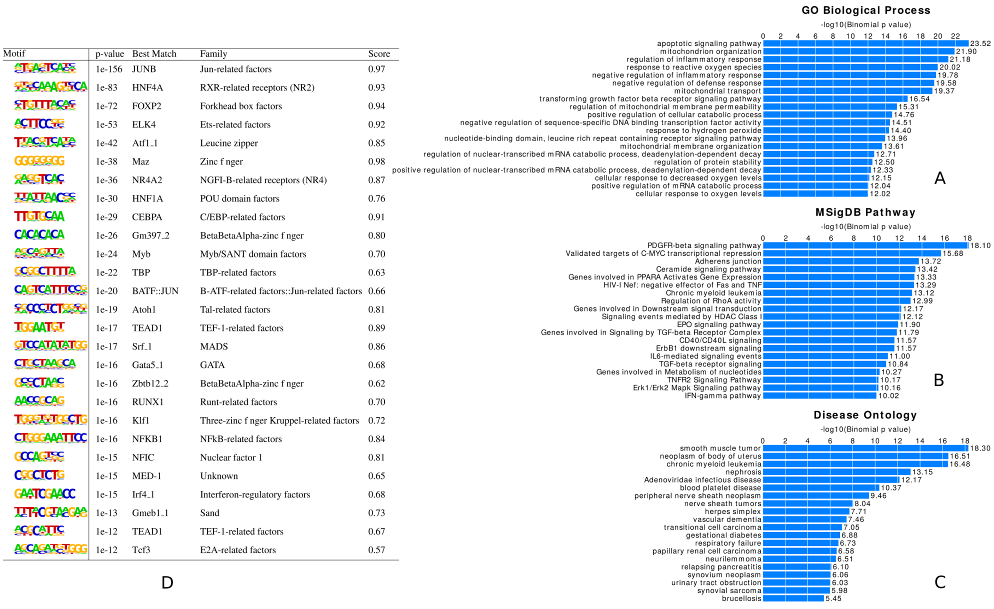

Motif	p-value	Best Match	Family	Score
	1e-156	JUNB	Jun-related factors	0.97
	1e-83	HNF4A	RXR-related receptors (NR2)	0.93
	1e-72	FOXP2	Forkhead box factors	0.94
	1e-53	ELK4	Ets-related factors	0.92
	1e-42	Atf1_1	Leucine zipper	0.85
	1e-38	Maz	Zinc f nger	0.98
	1e-36	NR4A2	NGFI-B-related receptors (NR4)	0.87
	1e-30	HNF1A	POU domain factors	0.76
	1e-29	CEBPA	C/EBP-related factors	0.91
	1e-26	Gm397_2	BetaBetaAlpha-zinc f nger	0.80
	1e-24	Myb	Myb/SANT domain factors	0.70
	1e-22	TBP	TBP-related factors	0.63
	1e-20	BATF::JUN	B-ATF-related factors::Jun-related factors	0.66
	1e-19	Atoh1	Tal-related factors	0.81
	1e-17	TEAD1	TEF-1-related factors	0.89
	1e-17	Srf_1	MADS	0.86
	1e-16	Gata5_1	GATA	0.68
	1e-16	Zbtb12_2	BetaBetaAlpha-zinc f nger	0.62
	1e-16	RUNX1	Runt-related factors	0.70
	1e-16	Klf1	Three-zinc f nger Kruppel-related factors	0.72
	1e-16	NFKB1	NFkB-related factors	0.84
	1e-15	NFIC	Nuclear factor 1	0.81
	1e-15	MED-1	Unknown	0.65
	1e-15	Irf4_1	Interferon-regulatory factors	0.68
	1e-13	Gmeb1_1	Sand	0.73
	1e-12	TEAD1	TEF-1-related factors	0.67
	1e-12	Tcf3	E2A-related factors	0.57

D

Figure S20: Functional and motif analysis of the DECRES NiA enhancers on cell line HepG2. A,B,C: Top 20 enriched biological processes, pathways, and diseases, respectively, in the NiA enhancers. D: Enriched *de novo* motifs in the NiA enhancer regions. Column 4: families of best-matched TFs. Column 5: best match scores.

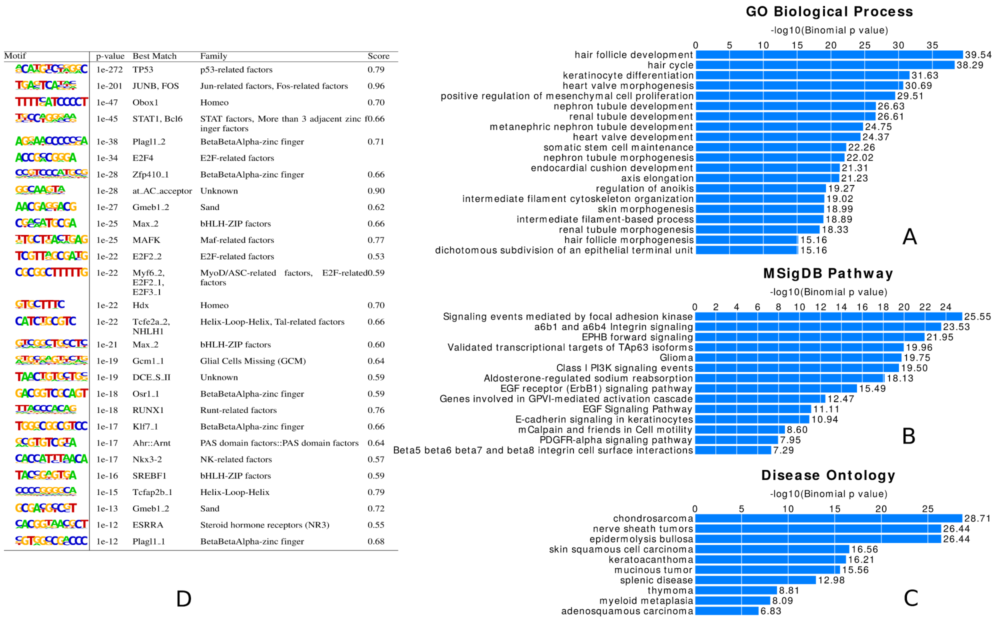

Motif	p-value	Best Match	Family	Score
	1e-272	TP53	p53-related factors	0.79
	1e-201	JUNB, FOS	Jun-related factors, Fos-related factors	0.96
	1e-47	Obox1	Homeo	0.70
	1e-45	STAT1, Bcl6	STAT factors, More than 3 adjacent zinc f0.66 inger factors	
	1e-38	Plagl1_2	BetaBetaAlpha-zinc finger	0.71
	1e-34	E2F4	E2F-related factors	
	1e-28	Zfp410_1	BetaBetaAlpha-zinc finger	0.66
	1e-28	at_AC_acceptor	Unknown	0.90
	1e-27	Gmeb1_2	Sand	0.62
	1e-25	Max_2	bHLH-ZIP factors	0.66
	1e-25	MAFK	Maf-related factors	0.77
	1e-22	E2F2_2	E2F-related factors	0.53
	1e-22	Myf6_2, E2F2_1, E2F3_1	MyoD/ASC-related factors, E2F-related0.59 factors	
	1e-22	Hdx	Homeo	0.70
	1e-22	Tcfe2a_2, NHLH1	Helix-Loop-Helix, Tal-related factors	0.66
	1e-21	Max_2	bHLH-ZIP factors	0.60
	1e-19	Gcm1_1	Glial Cells Missing (GCM)	0.64
	1e-19	DCE_S_II	Unknown	0.59
	1e-18	Osr1_1	BetaBetaAlpha-zinc finger	0.59
	1e-18	RUNX1	Runt-related factors	0.76
	1e-17	Klf7_1	BetaBetaAlpha-zinc finger	0.66
	1e-17	Ahr::Arnt	PAS domain factors::PAS domain factors	0.64
	1e-17	Nkx3-2	NK-related factors	0.57
	1e-16	SREBF1	bHLH-ZIP factors	0.59
	1e-15	Tcfap2b_1	Helix-Loop-Helix	0.79
	1e-13	Gmeb1_2	Sand	0.72
	1e-12	ESRRA	Steroid hormone receptors (NR3)	0.55
	1e-12	Plagl1_1	BetaBetaAlpha-zinc finger	0.68

D

Figure S21: Functional and motif analysis of the DECRES genome-wide predictions on cell line HMEC. A,B,C: Top enriched biological processes,pathways and diseases, respectively, in the predicted cell-specific CRRs. D: Enriched *de novo* motifs in the predicted cell specific CRRs. Column 4: families of best-matched TFs. Column 5: best match scores.

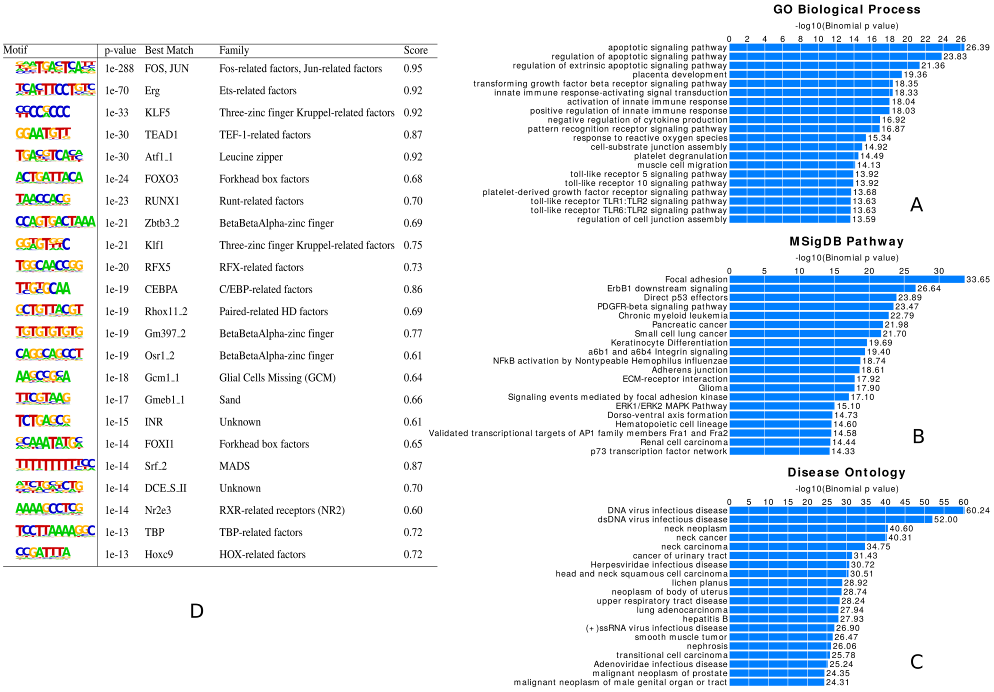

Motif	p-value	Best Match	Family	Score
	1e-288	FOS, JUN	Fos-related factors, Jun-related factors	0.95
	1e-70	Erg	Ets-related factors	0.92
	1e-33	KLF5	Three-zinc finger Kruppel-related factors	0.92
	1e-30	TEAD1	TEF-1-related factors	0.87
	1e-30	Atf1_1	Leucine zipper	0.92
	1e-24	FOXO3	Forkhead box factors	0.68
	1e-23	RUNX1	Runt-related factors	0.70
	1e-21	Zbtb3_2	BetaBetaAlpha-zinc finger	0.69
	1e-21	Klf1	Three-zinc finger Kruppel-related factors	0.75
	1e-20	RFX5	RFX-related factors	0.73
	1e-19	CEBPA	C/EBP-related factors	0.86
	1e-19	Rhox11_2	Paired-related HD factors	0.69
	1e-19	Gm397_2	BetaBetaAlpha-zinc finger	0.77
	1e-19	Osr1_2	BetaBetaAlpha-zinc finger	0.61
	1e-18	Gcm1_1	Glial Cells Missing (GCM)	0.64
	1e-17	Gmeb1_1	Sand	0.66
	1e-15	INR	Unknown	0.61
	1e-14	FOXI1	Forkhead box factors	0.65
	1e-14	Srf_2	MADS	0.87
	1e-14	DCE_S_II	Unknown	0.70
	1e-14	Nr2e3	RXR-related receptors (NR2)	0.60
	1e-13	TBP	TBP-related factors	0.72
	1e-13	Hoxc9	HOX-related factors	0.72

D

Figure S22: Functional and motif analysis of the DECRES NiA enhancers on cell line HMEC. A,B,C: Top 20 enriched biological processes, pathways, and diseases, respectively, in the NiA enhancers. D: Enriched *de novo* motifs in the NiA enhancer regions. Column 4: families of best-matched TFs. Column 5: best match scores.

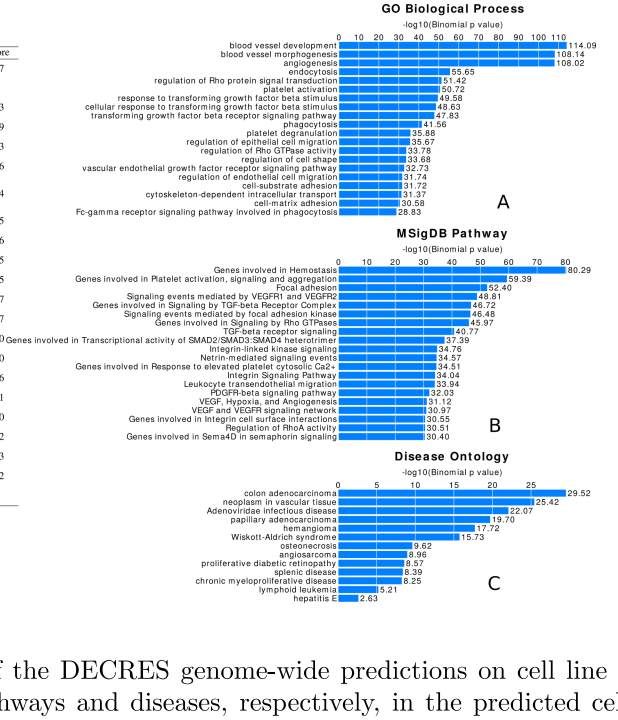

Motif	p-value	Best Match	Family	Score
	1e-999	JUNB, JUND, FOS, FOSL2	Jun-related factors, Fos-related factors	0.97
	1e-689	FEV, FLI1	Ets-related factors	0.93
	1e-142	ELF5	Ets-related factors	0.69
	1e-64	SOX9	SOX-related factors	0.93
	1e-48	GATA3, Gata4, Gata1	GATA-type zinc fingers	0.96
	1e-44	Foxa2, FOXA1	Forkhead box factors	0.74
	1e-43	SPIB	Ets-related factors	0.65
	1e-35	Mycn	bHLH-ZIP factors	0.96
	1e-31	Foxa2	Forkhead box factors	0.75
	1e-30	EHF	Ets-related factors	0.55
	1e-29	Mafb	Maf-related factors	0.77
	1e-24	Hic1_1	BetaBetaAlpha-zinc finger	0.67
	1e-23	Gata5_1	GATA-type zinc fingers	0.70
	1e-23	EHF	Ets-related factors	0.70
	1e-21	NFIC	Nuclear factor 1	0.86
	1e-19	Nkx3-1_1	NK-related factors	0.71
	1e-18	E2F6	E2F-related factors	0.60
	1e-15	Tcfap2b_2	Helix-Loop-Helix	0.72
	1e-13	MZF1	More than 3 adjacent zinc finger factors	0.63
	1e-12	SMAD2::SMAD3::SMAD4	SMAD factors	0.62

D

Figure S23: Functional and motif analysis of the DECRES genome-wide predictions on cell line HUVEC. A,B,C: Top enriched biological processes,pathways and diseases, respectively, in the predicted cell-specific CRRs. D: Enriched *de novo* motifs in the predicted cell specific CRRs. Column 4: families of best-matched TFs. Column 5: best match scores.

Motif	p-value	Best Match	Family	Score
	1e-382	JUNB, FOS	Jun-related factors, Fos-related factors	0.96
	1e-230	Erg	Ets-related factors	0.96
	1e-42	bZIP CREB/G-box-like subclass	CREB-related factors	0.91
	1e-39	GC-box, KLF5	Three-zinc finger Kruppel-related factors	0.89
	1e-32	FOXD1	Forkhead box factors	0.68
	1e-32	NFKB1	NFkB-related factors	0.79
	1e-31	RUNX1	Runt-related factors	0.73
	1e-31	Gata1	GATA-type zinc fingers	0.94
	1e-27	Nkx2-4	NK-related factors	0.57
	1e-27	Mafb	Maf-related factors	0.67
	1e-24	Crx	Paired-related HD factors	0.74
	1e-24	Myog, Tcf12	MyoD/ASC-related factors, E2A-related factors	0.93
	1e-23	NFATC2	NFAT-related factors	0.63
	1e-23	Hand1::Tcfe2a, NFIC	Tal-related factors::E2A-related factors, Nuclear factor 1	0.69
	1e-21	ARNT::HIF1A	PAS domain factors::PAS domain factors	0.75
	1e-21	Mybl1_1	Myb/SANT domain factors	0.71
	1e-21	Rfxdc2_1	RFX-related factors	0.78
	1e-19	Srf_2	MADS	0.87
	1e-18	Gm397_2	BetaBetaAlpha-zinc finger	0.72
	1e-17	Isgf3g_2	Interferon-regulatory factors	0.67
	1e-16	Zfp161_2	BetaBetaAlpha-zinc finger	0.69
	1e-16	at_AC_acceptor	Unknown	0.61
	1e-13	Rxra	RXR-related receptors (NR2)	0.55
	1e-12	Esrra_2	Hormone-nuclear receptor	0.68
	1e-12	Irf3_2	Interferon-regulatory factors	0.61

D

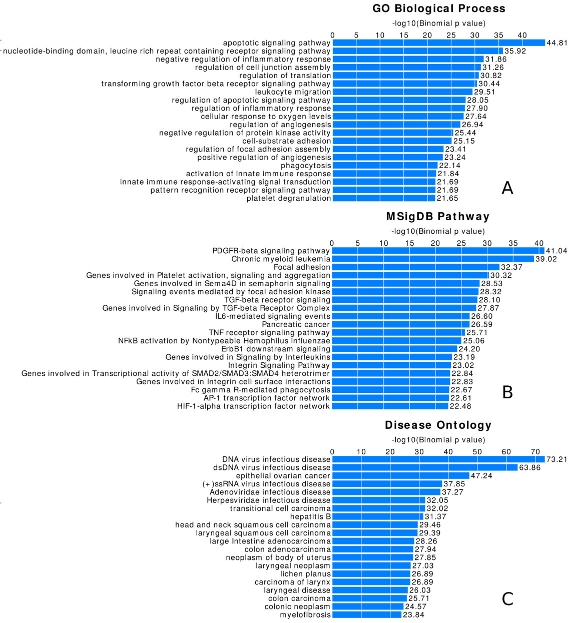

Figure S24: Functional and motif analysis of the DECRES NiA enhancers on cell line HUVEC. A,B,C: Top 20 enriched biological processes, pathways, and diseases, respectively, in the NiA enhancers. D: Enriched *de novo* motifs in the NiA enhancer regions. Column 4: families of best-matched TFs. Column 5: best match scores.

Motif	p-value	Best Match	Family	Score
	1e-2311	Gata6_1	GATA-type zinc fingers	0.94
	1e-418	FOS, FOSL2, JUNB, JUND	Fos-related factors, Jun-related factors	0.98
	1e-327	KLF5	Three-zinc finger Kruppel-related factors	0.92
	1e-189	Stat5a::Stat5b	STAT factors	0.94
	1e-187	Sfpi1_1	Ets-related factors	0.95
	1e-123	NFYB, NFYA	Heteromeric CCAAT-binding factors	0.91
	1e-122	Smad3_1	SMAD factors	0.65
	1e-120	Arnt, USF1, Mycn	PAS domain factors, bHLH-ZIP factors	0.93
	1e-117	Mafb_1	Maf-related factors	0.61
	1e-101	Stat6	STAT factors	0.71
	1e-99	YY1	More than 3 adjacent zinc finger factors	0.68
	1e-97	Zic1_2	BetaBetaAlpha-zinc finger	0.59
	1e-93	Glis2_1	BetaBetaAlpha-zinc finger	0.64
	1e-90	Zbtb12_1	BetaBetaAlpha-zinc finger	0.61
	1e-88	POU2F2	POU domain factors	0.69
	1e-84	at_AC_acceptor	Unknown	0.65
	1e-83	Myb	Myb/SANT domain factors	0.84
	1e-80	Smad3_1	SMAD factors	0.62
	1e-77	Zic2_2, Zic1_2	BetaBetaAlpha-zinc finger	0.62
	1e-65	Bcl6	More than 3 adjacent zinc finger factors	0.57
	1e-64	Zic2_2	BetaBetaAlpha-zinc finger	0.69
	1e-60	ETS_class	Ets-related factors	0.66
	1e-56	RUNX1	Runt-related factors	0.78
	1e-55	TEAD1	TEF-1-related factors	0.69
	1e-55	Hdx	Homeo	0.73
	1e-48	Pou6f1_1	POU domain factors	0.62
	1e-42	Osr1_2	BetaBetaAlpha-zinc finger	0.61
	1e-40	E2F2_2, E2F3_2	E2F-related factors	0.67
	1e-21	Rhox11_2, Rhox11_1	Paired-related HD factors	0.68
	1e-20	Dmbx1	Paired-related HD factors	0.58

D

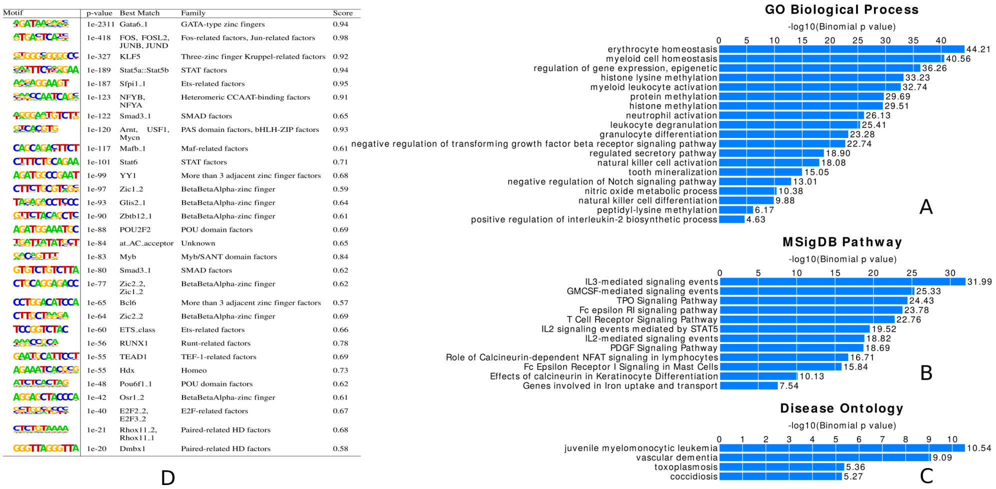

Figure S25: Functional and motif analysis of the DECRES genome-wide predictions on cell line K562. A,B,C: Top enriched biological processes,pathways and diseases, respectively, in the predicted cell-specific CRRs. D: Enriched *de novo* motifs in the predicted cell specific CRRs. Column 4: families of best-matched TFs. Column 5: best match scores.

Motif	p-value	Best Match	Family	Score
	1e-203	JUNB, FOSL2	Jun-related factors, Fos-related factors	0.98
	1e-109	Sfpi1_1, ELF1, Erg, FLI1	Ets-related factors	0.93
	1e-104	Gata1	GATA-type zinc fingers	0.94
	1e-61	KLF5, SP1	Three-zinc finger Kruppel-related factors	0.97
	1e-44	Atf1_1, Jundm2_1	(Leucine zipper), CREB-related factors, Jun-related factors	0.91
	1e-43	TRP(MYB)_class	Myb/SANT domain factors	0.85
	1e-38	USF1	bHLH-ZIP factors	0.96
	1e-34	bZIP_cEBP-like_subclass	C/EBP-related factors	0.67
	1e-33	NFYB	Heteromeric CCAAT-binding factors	0.91
	1e-29	CTCF	More than 3 adjacent zinc finger factors	0.85
	1e-28	Stat5a::Stat5b, STAT1	STAT factors	0.91
	1e-28	Foxo1	Forkhead box factors	0.75
	1e-20	Pou2f2	POU domain factors	0.68
	1e-20	Forkhead_class	Forkhead box factors	0.76
	1e-19	Pbx1	TALE-type homeo domain factors	0.64
	1e-18	Irf4_1	Interferon-regulatory factors	0.68
	1e-16	Tcf3_2	E2A-related factors	0.55
	1e-16	SMAD2::SMAD3::SMAD4	SMAD factors	0.71
	1e-12	Hmbox1	POU domain factors	0.56
	1e-12	NFYB	Heteromeric CCAAT-binding factors	0.65

D

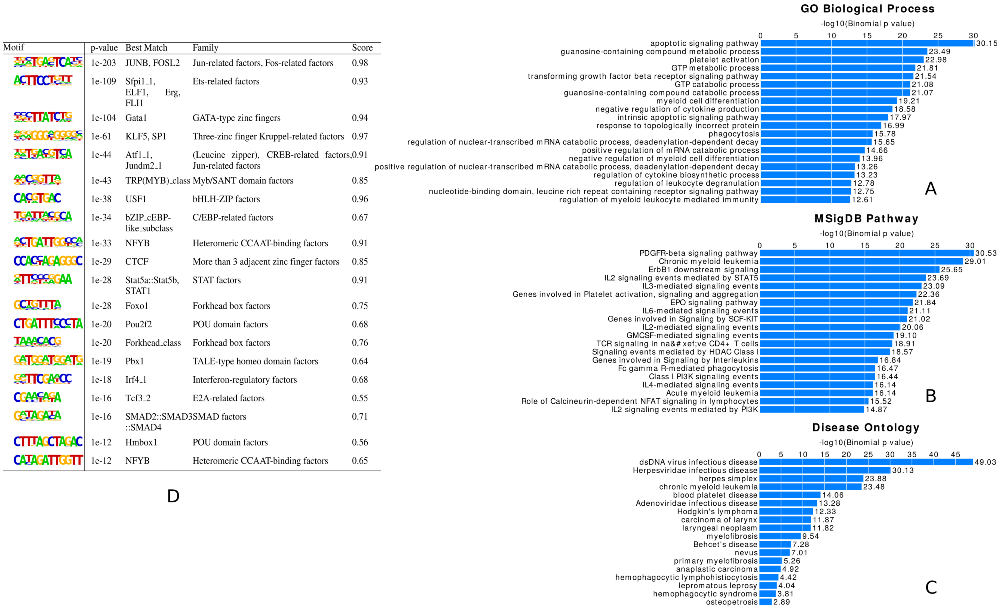

Figure S26: Functional and motif analysis of the DECRES NiA enhancers on cell line K562. A,B,C: Top 20 enriched biological processes, pathways, and diseases, respectively, in the NiA enhancers. D: Enriched *de novo* motifs in the NiA enhancer regions. Column 4: families of best-matched TFs. Column 5: best match scores.

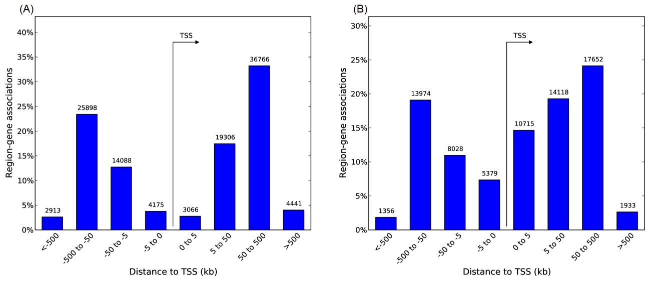

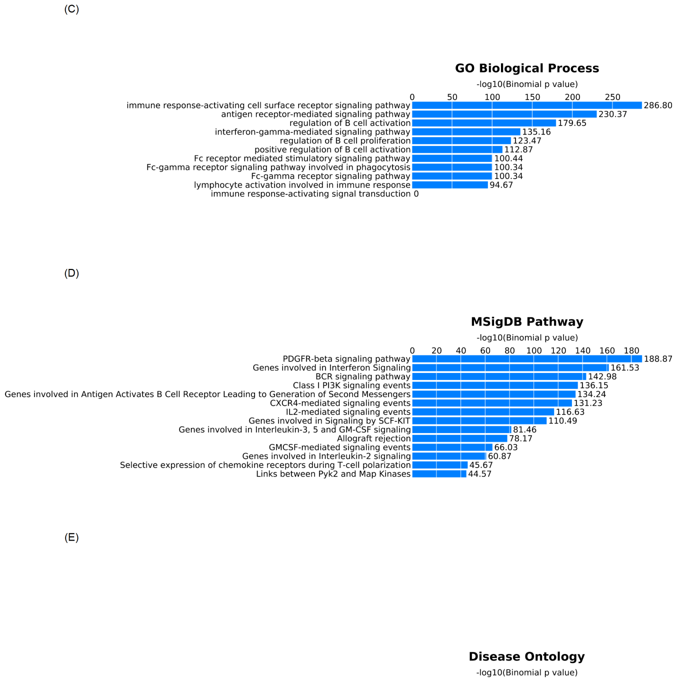

(F)

Motif	p-value	Best Match	Family	Score
	1e-1757	IRF1	Helix-turn-helix domains	0.955
	1e-702	Sfpi1	Helix-turn-helix domains	0.933
	1e-504	NFkB-p65	Immunoglobulin fold	0.972
	1e-371	RUNX1	Immunoglobulin fold	0.973
	1e-163	POU2F2	Helix-turn-helix domains	0.935
	1e-150	IRF4	Helix-turn-helix domains	0.843
	1e-129	BATF::JUN	Basic leucine zipper factors (bZIP)	0.939
	1e-100	CHR	Not available	0.671
	1e-89	Mef2a	alpha-Helices exposed by beta-structures	0.922
	1e-71	Meis1	Helix-turn-helix domains	0.764
	1e-54	MyoD	MyoD / ASC-related factors	0.818
	1e-33	NKX3-1	Helix-turn-helix domains	0.604
	1e-24	Sox17	Other all-alpha-helical DNA-binding domains	0.673
	1e-22	Zfp281	Zinc-coordinating DNA-binding domains	0.920
	1e-22	Rfx4	Helix-turn-helix domains	0.823
	1e-18	Osr2	Zinc-coordinating DNA-binding domains	0.594

Figure S27: Functional and motif analysis of the Combined genome-wide predictions on cell line GM12878. A: Distance from the predicted A-Es to gene TSSs. B: Distance from the predicted A-Ps to gene TSSs. C,D,E: Top 20 enriched biological processes, pathways, and diseases, respectively, in the predicted cell-specific CRRs. F: Enriched *de novo* motifs in the predicted cell specific CRRs. Column 4: families of best-matched TFs. Column 5: best match scores.

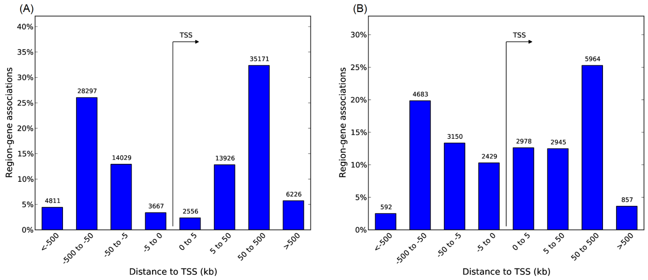

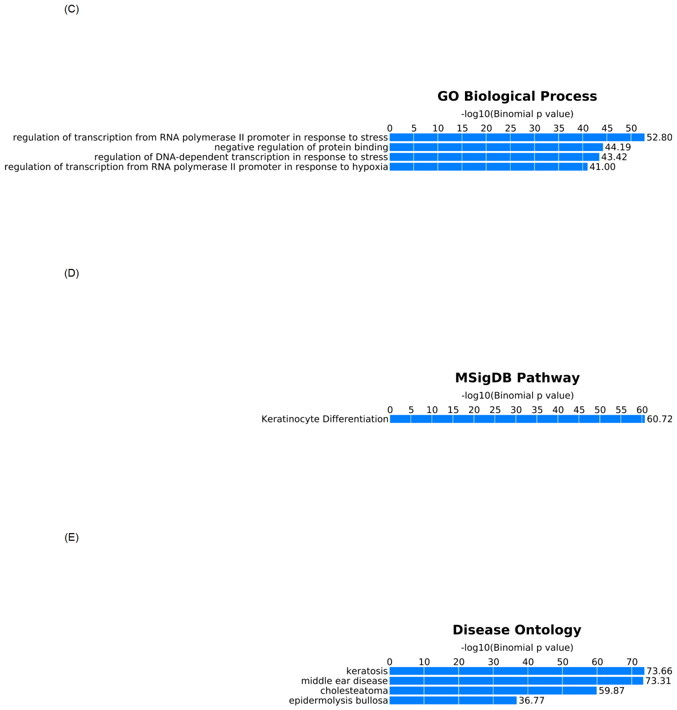

(F)

Motif	*p*-value	Best Match	Family	Score
GATGAGTCAT	1e-6754	BATF	Basic leucine zipper factors (bZIP)	0.987
TATTGTGCAATC	1e-564	CEBPA	C/EBP-related	0.974
AGGAATGC	1e-464	TEAD	Helix-turn-helix domains	0.966
AGGGCGTGGC	1e-399	KLF5	Zinc-coordinating DNA-binding domains	0.962
CCCTGAGGCAAT	1e-280	AP-2gamma	Zinc-coordinating DNA-binding domains	0.949
TGTAATCTGATA	1e-267	Six1	Helix-turn-helix domains	0.925
TGACATTTCCCC	1e-257	Nur77	Zinc-coordinating DNA-binding domains	0.900
GATGACGTCA	1e-243	Atf1	Basic leucine zipper factors (bZIP)	0.941
TGGCAATTTGCC	1e-233	Tlx?	Not available	0.883
TTATATAAACAA	1e-222	Forkhead_-class	Not available	0.940
CCTCTGGCAA	1e-169	NF1-halfsite	Zinc-coordinating DNA-binding domains	0.863
TGATAATATC	1e-156	GATA	Zinc-coordinating DNA-binding domains	0.913
GATAATTATC	1e-109	GATA	Zinc-coordinating DNA-binding domains	0.861
TAGTCACTGAGT	1e-84	Nkx3.1	Helix-turn-helix domains	0.611
CATAAATC	1e-79	Hoxc9	Helix-turn-helix domains	0.904
GAGACTAATGGG	1e-73	Isl1	Helix-turn-helix domains	0.691
CTGTGCCAGA	1e-73	Hand1::Tcfe2a	Twist-like factors	0.776
TACGCTGGACAA	1e-51	NFATC2	Immunoglobulin fold	0.633
GCCCTACCTTAA	1e-46	Zfp128	Zinc-coordinating DNA-binding domains	0.580
CCATTCTGAGTA	1e-45	STAT5	Immunoglobulin fold	0.611

Figure S28: Functional and motif analysis of the Combined genome-wide predictions on cell line HelaS3. A: Distance from the predicted A-Es to gene TSSs. B: Distance from the predicted A-Ps to gene TSSs. C,D,E: Top 20 enriched biological processes, pathways, and diseases, respectively, in the predicted cell-specific CRRs. F: Enriched *de novo* motifs in the predicted cell specific CRRs. Column 4: families of best-matched TFs. Column 5: best match scores.

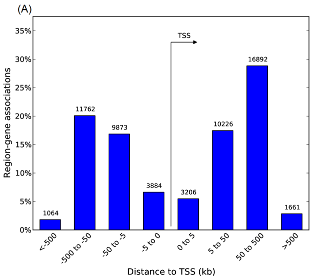

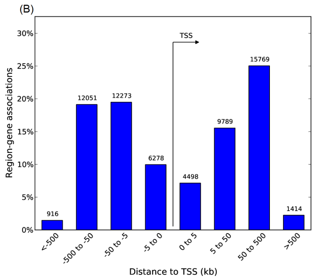

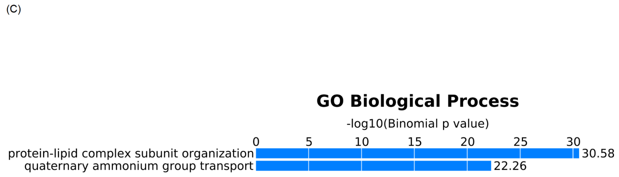

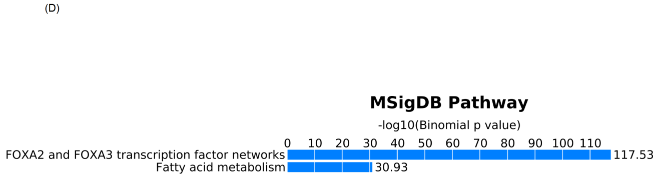

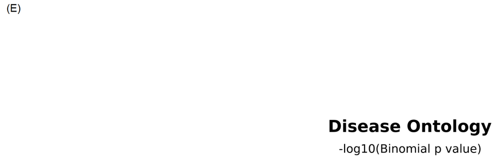

(F)

Motif	*p*-value	Best Match	Family	Score
GTCCAAAGTCCA	1e-1086	HNF4A	Zinc-coordinating DNA-binding domains	0.968
GAGTAAACAG	1e-423	Fox:Ebox	Helix-turn-helix domains	0.904
TTAATGATTAAC	1e-352	Hnf1	Helix-turn-helix domains	0.949
CTGACCTTGACC	1e-101	Nr5a2	Zinc-coordinating DNA-binding domains	0.876
GCAGCAATCTGA	1e-87	Six1	Helix-turn-helix domains	0.592
CATTGCTGAGGC	1e-74	MafA	Basic leucine zipper factors (bZIP)	0.632
TTGTGCAA	1e-68	CEBPA	C/EBP-related	0.901
AGGTCAGC	1e-63	Nuclear_-Receptor_-class	Not available	0.786
ATACTGCGCTTT	1e-61	Pou6f1	Helix-turn-helix domains	0.576
CCAGGGAGGGGC	1e-60	KLF5	Zinc-coordinating DNA-binding domains	0.875
TCACTGCTAG	1e-56	T	Basic helix-loop-helix factors (bHLH)	0.653
ACGCCCACGGAG	1e-49	Egr2	Zinc-coordinating DNA-binding domains	0.691
CAGGAAAGCT	1e-48	EWS:ERG-fusion	Helix-turn-helix domains	0.744
GGCTGCCGGC	1e-48	Tlx?	Not available	0.658
GGCCCCGGGC	1e-47	Tcfap2e	Basic domains	0.715
CGGGAAATGC	1e-43	NFKB1	Immunoglobulin fold	0.733
GTCTTAGCAATC	1e-42	Rfx3	Helix-turn-helix domains	0.670
CTAGGCAAGGGA	1e-42	at_AC_acceptor	Not available	0.575
CGTGGAAAAT	1e-41	NFATC2	Immunoglobulin fold	0.892
GGAAGCTCGAAC	1e-40	DCE_S_I	Not available	0.637

Figure S29: Functional and motif analysis of the Combined genome-wide predictions on cell line HepG2. A: Distance from the predicted A-Es to gene TSSs. B: Distance from the predicted A-Ps to gene TSSs. C,D,E: Top 20 enriched biological processes, pathways, and diseases, respectively, in the predicted cell-specific CRRs. F: Enriched *de novo* motifs in the predicted cell specific CRRs. Column 4: families of best-matched TFs. Column 5: best match scores.

(A)

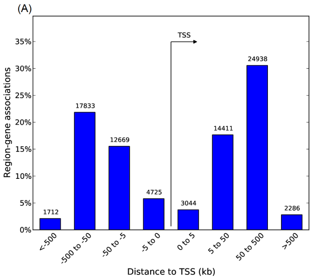

(B)

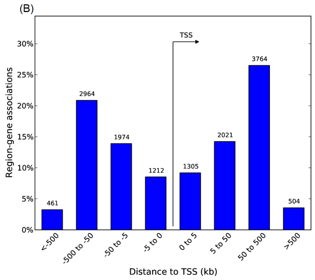

(C)

(D)

(E)

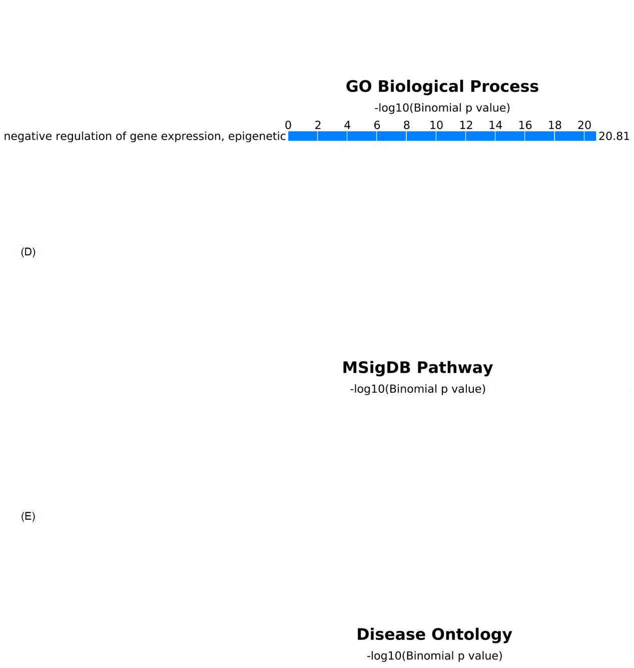

(F)

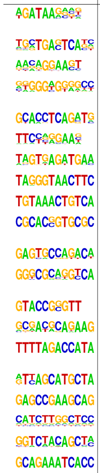

Motif	*p*-value	Best Match	Family	Score
	1e-2706	Gata2	Zinc-coordinating DNA-binding domains	0.980
	1e-630	Bach2	NF-E2-like factors	0.974
	1e-238	Sfpi1	Helix-turn-helix domains	0.921
	1e-227	KLF5	Zinc-coordinating DNA-binding domains	0.921
	1e-202	CREB	CREB-like factors	0.600
	1e-176	STAT5	Immunoglobulin fold	0.948
	1e-175	Six1	Helix-turn-helix domains	0.581
	1e-173	Spz1	Not available	0.667
	1e-170	Myb	Helix-turn-helix domains	0.763
	1e-166	Zfp161	Zinc-coordinating DNA-binding domains	0.602
	1e-163	Tbox:Smad	Not available	0.805
	1e-140	RXR	Zinc-coordinating DNA-binding domains	0.711
	1e-133	Nkx3-1	Helix-turn-helix domains	0.546
	1e-130	DCE_S_I	Not available	0.613
	1e-128	Zfp187	Zinc-coordinating DNA-binding domains	0.593
	1e-126	Pou2f3	Helix-turn-helix domains	0.639
	1e-121	DCE_S_I	Not available	0.611
	1e-116	E2F2	Helix-turn-helix domains	0.632
	1e-111	DCE_S_II	Not available	0.614
	1e-109	Hdx	Helix-turn-helix domains	0.749

Figure S30: Functional and motif analysis of the Combined genome-wide predictions on cell line K562. A: Distance from the predicted A-Es to gene TSSs. B: Distance from the predicted A-Ps to gene TSSs. C,D,E: Top 20 enriched biological processes, pathways, and diseases, respectively, in the predicted cell-specific CRRs. F: Enriched *de novo* motifs in the predicted cell specific CRRs. Column 4: families of best-matched TFs. Column 5: best match scores.

References

[1] B. Arnett, P. Soisson, B.S. Ducatman, and P. Zhang. Expression of CAAT enhancer binding protein beta (C/EBP beta) in cervix and endometrium. *Molecular Cancer*, 2:21, 2003.

[2] M. Beger, K. Butz, C. Denk, T. Williams, H.C. Hurst, and F. Hoppe-Seyler. Expression pattern of AP-2 transcription factors in cervical cancer cells and analysis of their influence on human papillomavirus oncogene transcription. *Journal of Molecular Medicine*, 79(5-6):314–320, 2001.

[3] R.H. Costa, V.V. Kalinichenko, A.X. Holterman, and X. Wang. Transcription factors in liver development, differentiation, and regeneration. *Hepatology*, 38(6):1331–1347, 2003.

[4] A. Dev, S. Iyer, B. Razani, and G. Cheng. NF-κB and innate immunity. *Current Topics in Microbiology and Immunology*, 349:115–143, 2011.

[5] J.D. Fleming, G. Pavesi, P. Benatti, C. Imbriano, R. Mantovani, and K. Struhl. NF-Y coassociates with FOS at promoters, enhancers, repetitive elements, and inactive chromatin regions, and is stereo-positioned with growth-controlling transcription factors. *Genome Research*, 23(8):1195–1209, 2013.

[6] D. Kleftogiannis, P. Kalnis, and V.B. Bajic. DEEP: A general compuational framework for predicting enhancers. *Nucleic Acids Research*, 43(1):e6, 2015.

[7] A.K. Linnemann, H. O'Geen, S. Keles, P.J. Farnham, and E.H. Bresnick. Genetic framework for GATA factor function in vascular biology. *Proceedings of the National Academy of Sciences*, 108(33):13641–13646, 2011.

[8] R. Lopez, E. Garrido, G. Vazquez, P. Pina, C. Perez, I. Alvarado, and M. Salcedo. A subgroup of HOX Abd-B gene is differentially expressed in cervical cancer. *International Journal Gynecological Cancer*, 16(3):1289–1296, 2006.

[9] A.L. Malt, J. Cagliero, K. Legent, J. Silber, A. Zider, and D. Flagiello. Alteration of TEAD1 expression levels confers apoptotic resistance through the transcriptional up-regulation of Livin. *PLoS One*, 7(9):e45498, 2012.

[10] E.V. Mityushova, N.D. Aksenov, and I.I. Marakhova. STAT5 in regulation of chronic leukemia K562 cell proliferation: Inhibitory effect of WHI-P131. *Cell and Tissue Biology*, 4(1):63–69, 2010.

[11] T. Okuda, M. Nishimura, M. Nakao, and Y. Fujita. RUNX1/AML1: A central player in hematopoiesis. *International Journal of Hematology*, 74(3):252–257, 2001.

[12] G.V. Samant, M.O. Schupp, M. Francois, S. Moleri, R.K. Kothinti, C.Z. Chun, I. Sinha, S. Sellars, N. Leigh, K. Pramanik, M.A. Horswill, I. Remadevi, K. Li, G.A. Wilkinson, N.M. Tabatabai, M. Beltrame, P. Koopman, and R. Ramchandran. Sox factors transcriptionally regulate ROBO4 gene expression in developing vasculature in zebrafish. *The Journal of Biological Chemistry*, 286:30740–30747, 2011.

[13] H. Schrem, J. Klempnauer, and J. Borlak. Liver-enriched transcription factors in liver function and development. Part II: The C/EBPs and D site-binding protein in cell cycle control, carcinogenesis, circadian gene regulation, liver regeneration, apoptosis, and liver-specific gene regulation. *Pharmacological Reviews*, 56(2):291–330, 2004.

[14] C.C. Thornton, F. Al-Rashed, D. Calay, G.M. Birdsey, A. Bauer, H. Mylroie, B.J. Morley, A.M. Randi, D.O. Haskard, J.J. Boyle, and J.C. Mason. Methotrexate-mediated activation of an AMPK-CREB-dependent pathway: A novel mechanism for vascular protection in chronic systemic inflammation. *Annals of the Rheumatics Diseases*, 75(2):439–448, 2015.

[15] P. Wang, J. Xu, and C. Zhang. CREB, a possible upstream regulator of Bcl-2 in trichosanthin-induced HeLa cell apoptosis. *Molecular Biology Reports*, 37(4):1891–1896, 2010.

[16] Z. Wang, E.P. Bishop, and P.A. Burke. Expression profile analysis of the inflammatory response regulated by hepatocyte nuclear factor 4α. *BMC Genomics*, 12:128, 2011.

[17] H. Zhang, C. Liu, Z. Zha, B. Zhao, J. Yao, S. Zhao, Y. Xiong, Q. Lei, and K. Guan. TEAD transcription factors mediate the function of TAZ in cell growth and epithelial-mesenchymal transition. *The Journal of Biological Chemistry*, 284(20):13355–13362, 2009.

[18] J. Zhu, D.M. Giannola, Y. Zhang, A.J. Rivera, and S.G. Emerson. NF-Y cooperates with USF1/2 to induce the hematopoietic expression of HOXB4. *Blood*, 102(7):2420–2427, 2003.
